# Pharmaceutical Humanities and Narrative Pharmacy: An Emerging New Concept in Pharmacy

**DOI:** 10.3390/ph18010048

**Published:** 2025-01-03

**Authors:** Mita Banerjee, Thomas Efferth

**Affiliations:** 1 Department of English and Linguistics, Obama Institute for Transnational American Studies, Johannes Gutenberg University, Jakob Welder Weg 20, 55128 Mainz, Germany; 2 Department of Pharmaceutical Biology, Institute of Pharmaceutical and Biomedical Sciences, Johannes Gutenberg University, Staudinger Weg 5, 55128 Mainz, Germany

**Keywords:** bioethics, ethics, global threats, interdisciplinary research, natural products, sustainability, technology impact and risk assessment, narrative pharmacy, medical humanities

## Abstract

The complexity of our life experiences and the rapid progress in science and technology clearly necessitate reflections from the humanities. The ever-growing intersection between science and society fosters the emergence of novel interdisciplinary fields of research. During the past decade, Medical Humanities arose to meet the need to unravel hidden information beyond technology-driven and fact-based medicine. In the present paper, we put forward the hypothesis that there is a similar requirement to develop Pharmaceutical Humanities as an academic discipline within pharmacy and pharmaceutical biology. Based on Thomas Kuhn’s epistemological theory on the structure of scientific revolutions, one may argue that a paradigm change for Pharmaceutical Humanities might open new levels of insight. Many complex diseases (e.g., cancer, neurological diseases, and mental disorders) remain uncurable for many patients by current pharmacotherapies, and the old beaten paths in our therapeutic thinking may at least partly have to be left behind. By taking examples from Pharmaceutical Biology, we attempt to illustrate that the transdisciplinary dialogue with the humanities is fertile ground not only for enlarging our understanding of disease-related conditions but also for exploring new ways of combatting diseases. In this context, we discuss aspects related to traditional herbal medicine, fair access and benefit sharing of indigenous knowledge about medicinal plants, post-traumatic stress syndrome, the opioid crisis, stress myocardiopathy (broken heart syndrome), and global environmental pollution with microplastics. We also explore possibilities for a narrative turn in pharmacy. The urgent need for inter- and transdisciplinary solutions to pressing health-related problems in our society may create a scholarly atmosphere for the establishment of Pharmaceutical Humanities as a fruitful terrain to respond to the current demands of both science and society.

## 1. Introduction: The Benefit of Including the Humanities in Medicine and Beyond

In the age of biomedicine, we are increasingly confronted with problems that seem impossible to solve without the help of interdisciplinarity. From technologically assisted reproduction to decisions at the end of life, biomedicine provides us with technologies that enable us to change the course of human life in an unprecedented manner. However, the ever-increasing range of technologies provided by both medicine and the life sciences may confront us with decisions we have never had to make before. We are thus confronted with decision making at the interface of science and society: Not everything that is biomedically possible may also be desirable. In other words, science and society must go hand in hand to find solutions to situations that concern both the present and the future of human life. In recent years, this has become especially pronounced in the debates surrounding CRISPR/Cas9 [1].

Given the need for decision making at the intersection between science and society, it seems only natural that new, interdisciplinary fields have emerged over the last two decades. In this context, the Medical Humanities have been especially visible. During the past 10 years, the field of Medical Humanities has become increasingly established as a link between medicine and the humanities. Medical Humanities are a new discipline located between all medical disciplines and all disciplines from humanities and social sciences (bioethics, philosophy, cultural studies, anthropology, and sociology) and also arts (literature, theater, and film) [2,3]. The concept is to apply non-conform methods to unravel hidden information behind diseases and disease-causing conditions. More and more universities in the United States have been establishing master programs for graduate students in Medical Humanities [4]. A website of the Health Humanities Consortium offers a list of graduate programs in Medical and Health Humanities throughout the US (https://healthhumanitiesconsortium.com/publications/graduate-programs/, accessed on 28 December 2024). This shows that Medical Humanities are increasingly making inroads into medical education. However, this is a relatively recent trend. Penn State College was the first medical school to offer Medical Humanities courses as early as 1967; however, such programs still constitute an exception. This is true both in the US and worldwide [5]. The inclusion of the humanities through Medical Humanities programs is increasingly being seen as a way of enhancing sensitivity in doctors as well as doctor–patient communication [6,7].

As a novel discipline, Medical Humanities develop true interdisciplinary platforms as a novel and independent concept that is different from classical cross-disciplinary projects, where different disciplines jointly cooperate but each of these collaborating disciplines remains in its specific academic framework. By contrast, the Medical Humanities aim to generate novel knowledge not by simply merging established disciplines but by fostering the dialogue and critical discourse among them. Hence, a unique opportunity which is presented by Medical Humanities is the discussion with rather than about medical disciplines. Medical Humanities have been especially prominent in the teaching of medicine [8,9]. Proponents of the Medical Humanities have stressed the role of cultural texts such as literature, film, or painting in enhancing medical students’ understanding of the illness experience. The field of narrative medicine has emerged as a methodology closely connected to the Medical Humanities [10,11]. In narrative medicine workshops, which at some medical schools have become a mandatory part of the curriculum, future practitioners of medicine are invited to change perspectives [10,12]: by immersing themselves in literary narratives, films, or paintings about an illness, they adopt the perspective of the patient. This change in perspective can serve to enhance future doctors’ understanding of the practice of medicine [13]. Since medicine, in the clinical setting, is based on the fixed roles of doctor and patient, much can be gained from delving more deeply into specific requirements and expectations connected to these roles [14]. Studies have shown that cultural and artistic texts such as literary narratives can play a key role in this regard: As medical practitioners immerse themselves into the fictional universe of literary texts, they become estranged from their own everyday clinical setting [15]. As they read narratives that depict situations of care—often situations that do not feature an explicitly clinical setting—they are caused to reconsider what it means to care and to be cared for. Narrative medicine hence uses the methodology of estrangement, in order to deliberately suspend the knowledge that physicians have about medical practice [16,17]. At the same time, some critics have argued that the methodology of narrative medicine may not be without its own pitfalls: the primacy of narrative, they argue, puts an emphasis on verbal communication, potentially marginalizing non-verbal individuals; moreover, it may value narrative coherence over episodic conceptions of the self [18].

By rethinking medical knowledge in the safe space of literary analysis, doctors may become more aware of their own roles and the limitations of these roles. Crucially, studies have shown that training in narrative medicine and Medical Humanities, in general, can enhance doctor–patient communication [19]. Reading literary texts written from the perspective of the patient may foster empathy and may thus prove beneficial in communicating with patients in the clinical setting.

Re-integrating medical humanities into the humanities, in turn, can lead to a revival of humanistic models of explaining the world. There is no doubt that the (post)modern world is based on scientific principles of order and that there was a paradigm shift in the 18th century at the time of the Enlightenment. Humanistic (including religious) conceptions of the world were replaced by materialistic views of man and nature. Since the beginning of the 20th century, there has been an increasing importance of the individual over the collective, which is reflected today in biomedicine. The conceptualization of individualized precision medicine is an expression of current research approaches that have already gone back decades [20,21]. The principle of strengthening the individual within society is valid at least for western, democratic industrial nations, even as counteracting tendencies can be found everywhere in some autocratic forms of society. This shows once more that scientific and medical progress on the one hand and spiritual scientific reflection on the other hand can be separated only with difficulty. Science and society are inseparable, and it has been the role of the humanities, along with the social sciences, to reflect on the values and tenets that societies are based on. As these considerations show, Medical Humanities and narrative medicine have increasingly developed into a complex, highly interdisciplinary field at the intersection between medicine and the humanities. The narrativization of empirically collected data or lived life experiences can be a basis for the creation of common methodological approaches for a better understanding of the human being in his complex whole and of being human in today’s world. As the humanities are increasingly interested in collaborating with the natural sciences, it may be no coincidence that they should have turned to medicine as a natural ally [22]. As Cornelius Borck has suggested, medicine can itself be seen as being akin to the humanities, or it may even be grouped among the humanities as such [23]. Not only is medicine the largest discipline within the life sciences, but it may also possess characteristics, which make it particularly susceptible to input from the humanities. As outlined above, this may be especially true for the role of doctor–patient communication [24].

At the same time, the potential inherent in interdisciplinary work between the natural sciences and the humanities far exceeds the focus of the Medical Humanities [25]. In fact, there may be a danger inherent in grouping all studies which explore connections between the humanities and the life sciences simply under the rubric of Medical Humanities. Seen from this perspective, Medical Humanities may thus carry the risk of homogenizing both medicine and the life sciences in general. Medical Humanities are concerned with bringing narratives about the illness experience to the medical classroom [26]. Because they place central importance on the experience of the illness itself, they may at times run the risk of glossing over the specific nature of the illness, or the discipline within medicine that sets out to treat a particular condition. Generally, Medical Humanities are interested in the mutual dialogue between medicine and the humanities [7]. Such a discussion is closely related to the increasing use of biohumanities, which have explored the interplay between biosciences, philosophy, and history of science [27,28]. What is particularly noteworthy here is that universities are beginning to introduce academic programs which specifically link humanities and biology, such as a program at Carleton University in Canada (https://carleton.ca/bhum/humanities-biology/, accessed on 28 December 2024).

This paper argues that the potential for future interdisciplinary research which involves both the natural sciences and the humanities may by far exceed the Medical Humanities as a framework. We argue that this potential may only come to fruition if we take into account the specific disciplines within the natural sciences that the humanities are in dialogue with. For this reason, a recent study argues that it may be more productive to speak of biological humanities as an overarching framework that accounts for the role not only of medicine but also of other disciplines within the natural sciences in the dialogue with the humanities [25].

In this essay, we will explore the role of the Pharmaceutical Humanities as a subfield of the biological humanities. We will show in this article that the Pharmaceutical Humanities are highly productive not only owing to their link to medicine, but also to other disciplines within and beyond the natural sciences.

In addition to interventional therapy with drugs, clinical pharmacy also focuses on disease prevention and rehabilitation. Main tasks of pharmacy include

- Patient counseling and care to ensure safe and effective drug intake;
- Monitoring of compliance to ensure that patients correctly take their medication;
- Information and training of patients to ensure correct drug intake to avoid unwanted drug–drug interactions;
- Continuing and further education for other healthcare professionals;
- Patient visits: pharmacists participate in the daily visits of patients together with physicians.

Classical tasks in pharmacotherapy such as medication supply and preparation are complemented by non-pharmacological treatment approaches such as psychosocial care, physical therapies, and nutrition therapy. Hence, pharmacy aims to optimize disease management in a comprising and “holistic” manner.

This multitude of tasks illustrates how important it is that pharmacists need to constantly train and improve their empathy for the patient, reflect on the patient’s situation, and improve their communication skills. In this context, narrative pharmacy and Pharmaceutical Humanities are of utmost importance to better understand emotions, worries, fears, and personal life histories. Patients may feel left alone and misunderstood in a technocratic, machine-driven medical world. This situation may lead to frustration, non-compliance with sub-optimal therapy outcome, or even treatment failure. We argue that methods and techniques of Pharmaceutical Humanities are as important for pharmacists as the Medical Humanities are for physicians to better understand the mental crises and case histories of patients. This entire field is considerably understudied. It has been estimated that non-drug interventional topics make up only 40% of all clinical trials (i.e., application observations, post-authorization safety studies, cohort studies, and case-control studies).

In this context, it may be interesting to recall the different meanings of the terms “disease, illness, and sickness”. As outlined by J. Russell Teagarden [29], Bjørn Hofmann described disease as the pathophysiological, biochemical, and molecular mechanisms. Illness refers to the experiences a patient makes, and sickness is the response of the society or official health system to it [30]. Expressed in other words, you could say: a patient feels ill when he goes to the doctor and has a disease when he leaves him (according to Eric Cassel) [31].

**Figure 1:**
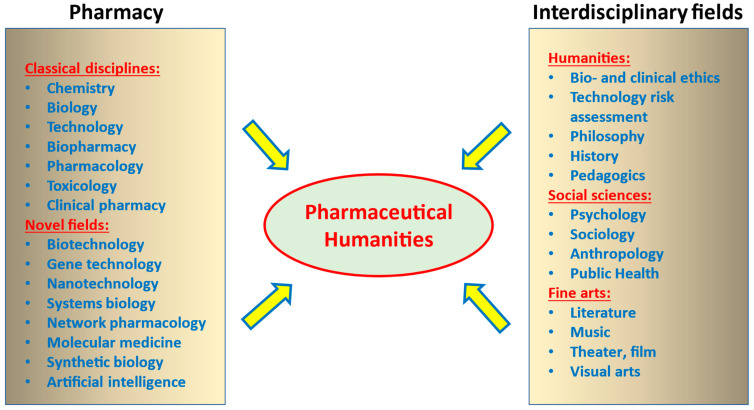
Pharmaceutical Humanities at the interface between natural sciences and humanities. Due to the tremendous advancement in the life sciences in general, the classical pharmaceutical sciences (chemistry, biology, technology, biopharmacy, pharmacology, toxicology, and clinical pharmacy) have been complemented by novel disciplines (biotechnology, gene therapy, nanotechnology, systems biology, network pharmacology, molecular medicine synthetic biology, artificial intelligence). The treatment of patients has always consisted of drug-based interventional and non-drug-based non-interventional methods. Therefore, the rethinking of the inclusion of humanistic expertise into pharmacy is obvious. There is an increasing body of evidence that these non-interventional interdisciplinary fields can contribute substantially to the therapeutic success of treating diseases. Examples are documented from the humanities (bio- and clinical ethics, technology risk assessment, philosophy, history, and pedagogics), the social sciences (psychology, sociology, anthropology, and public health), and fine arts (literature, music, theater, film, and visual arts). Because patients can benefit from all these diverse treatment approaches, we propose a stronger focus on the inclusion of non-interventional therapies rooting in non-classical pharmaceutical, interdisciplinary fields into natural science-based and drug-based interventional pharmacy by establishing the Pharmaceutical Humanities as a novel discipline.

In this vein, since medicine and pharmacy are separate yet related disciplines, Pharmaceutical Humanities can be distinguished from Medical Humanities. Pharmacy deals with its own specific topics that cannot be handled by a physician focusing on patient care. Typical pharmaceutical topics are the development, assessment, production, and selling of medications. As such, pharmacy represents an interdisciplinary scientific area combining diverse natural sciences (i.e., chemistry, biology, technology, pharmacology, toxicology, and physics) but also law, pharmacy history, and business administration. Thus, Pharmaceutical Humanities can be considered as an overlapping but clearly distinct field of interest from Medical Humanities. Moreover, the framework of the Pharmaceutical Humanities is of interest not only in terms of theorizing science but it also has great potential for application. Like only a few other disciplines, the pharmaceutical curriculum is itself highly interdisciplinary: The Pharmaceutical Humanities may, thus, be ideally suited for interdisciplinary dialogue with the humanities (Figure 1).

The beginning of the twenty-first century seems particularly suited for the emergence of new fields such as pharmaceutical biology and narrative pharmacy. This is particularly the case since in the more recent histories of their respective academic developments, the humanities and the life sciences have been converging: the humanities have undergone a material turn [32], while the life sciences have undergone a narrative turn [33,34]. In the course of their material turn, the humanities have paid renewed attention to the workings of the human body and the human brain in particular [35,36]. In the wake of this paradigm change, researchers have paid specific attention to the use of narrative (framing, narrative perspective, and use of metaphors) in the communication of scientific results [37]. Similarly, natural scientists have explored the role of metaphor and narrative for shaping scientific thought [38,39].

This convergence—the material turn of the humanities and the narrative turn in the life sciences—makes for the fact that at the present moment, the Pharmaceutical Humanities can unfold great interdisciplinary potential [40]. At the interface between pharmacy and the humanities, a wide variety of approaches and methodologies may appear, which have so far gone unnoticed. As long as we have focused only on the Medical Humanities, these new avenues for research may not have been visible as paradigms in their own right. However, in recent years, numerous studies in basic research have appeared, which can be seen as the focal point of a newly emerging scientific discipline: the Pharmaceutical Humanities. In this article, we provide not only an overview of different aspects of the Pharmaceutical Humanities, but we will also situate this new field in theoretical terms. We will especially discuss the theoretical location of newly emerging fields in the history of science. We will first discuss the relationship between life sciences and the humanities in general. We will then move on to an exploration of scientific progress and paradigm change. One of the questions addressed in this context is whether, with regard to theories of science, we can view the Pharmaceutical Humanities as an emerging new discipline. In the last section of this article, concrete examples of the range and the versatility of the Pharmaceutical Humanities will be provided.

## 2. Embedding the Pharmaceutical Humanities in a Historical Context

In order to take a closer look at the relationship between the natural sciences and the humanities, it is important to situate this discussion in the field of the philosophy of science. In this subchapter of our paper, our aim is to trace the historical developments—in both science and the humanities—which have led up to the present moment. This is important for understanding the split between the humanities and the natural sciences that occurred in the nineteenth century, and which the Pharmaceutical Humanities seek to bridge. Following this split into “two cultures” [41], each of these groups of disciplines took a different trajectory. This divergent development, in turn, can be seen as an obstacle to cross-cutting fields such as the Pharmaceutical Humanities. A discussion of Pharmaceutical Humanities as a current new field must hence take into account historical developments: in order to transcend the divide between life sciences and humanities, we must revisit the historical context in which it first occurred. As a field within the discipline of philosophy, the theory of science has explored, first and foremost, the “nature” of scientific knowledge as such [42,43]. The aim of a general theory of science is thus a universal description of all scientific fields, regardless of their specialization into particular disciplines. One of the central concerns of the theory of science is the role of methodologies. In line with its aim to describe science as such, it is less interested in particular, discipline-specific methodologies than in the existence and formation of methodologies as such. Different disciplines may thus differ in the particular methodologies they employ, but they converge in a common need for methodologies. These methodologies, in turn, are the subject of perpetual discussion: they are tested against other methodologies, and they may become obsolete and be superseded by new forms of experimentation and validation, particularly in tandem with the rise of new technologies. Researchers argue that the discussion among peers that constitute a scientific community is key to an understanding of how science is constituted [44]. Not incidentally, different forms of peer review are the backbone of quality standards, particularly in the natural sciences [45]. As part of and beyond peer review, the concept of verification and validation is central to an understanding of the nature of science itself. Here, the concept of falsification, the mandate that a given research result, obtained through the use of specific methodologies, can be falsified, has been said to be one of the central differences between the natural sciences and the humanities. The result of a scientific experiment can be falsified only through empirical review. The notion of falsification hence emerges from the disciplinary and methodological setup of the natural sciences [46], as opposed to the theoretical repertoire of the humanities [47,48]. Since the humanities do not constitute empirical sciences in the sense commonly used with regard to the natural sciences, the concept of falsification does not seem to apply to them. However, the humanities—as human sciences—are similarly based on the application of specific methods to a set of data. Hence, they share a central criterion that all sciences must strive for, namely the goal to achieve a measure of truth [49,50]. Yet, it is important to note in this context that “truth”—as a reality that can be objectively described—is subject to historical change. What researchers found to be “true” at one moment in time can subsequently be refuted or falsified, often with the help of new technologies. Such falsification, in turn, gives rise to scientific progress [51]. The goal of truth-seeking is thus common to both sciences and the humanities. The very distinction between these two groups of disciplines emerged only in the mid-19th century [52].

The question of “truth” is an important one in an epistemological philosophical theory of the 20th century called “constructivism” [53]. Each individual constructs their own reality on the basis of their own life experiences. Whether this individually constructed truth corresponds to absolute truth or whether there can be absolute truth at all remains an open question [54]. This difference in the definition of truth lies at the heart of the split between humanities and life sciences; it has been one of the obstacles against the interdisciplinary cooperation between these fields. The humanities as we know them today originated in the so-called artes liberales or liberal arts. They comprised the fields of literature, grammar, rhetoric, history, and logic. The 19th century then saw the emergence of classical philology [55], which was followed by the creation of philologies based on new languages (*neusprachliche Philologien*). The term *Geisteswissenschaften* (sciences of the mind) was coined only in the 19th century by Wilhelm Dilthey [56]. Over the past two decades, these *Geisteswissenschaften* developed into an increasingly diverse field, for which we use the Anglo-American term of the “humanities” today. As part of their development from philology into humanities, different disciplines—from history to law, sociology, political science, and even psychology—came to be grouped under the common umbrella of the humanities [57]. The inclusion of psychology as part of the humanities [58] already indicates the fissuring out of the dividing line between the natural sciences and the humanities: as the methodologies used in psychology are based on empirical methods to a large extent, the distinction between the empirical sciences and the humanities can be seen to be blurred.

In addition to the difference between empirical and non-empirical methodologies, the sciences and the humanities were separated by their perspective on the object which they sought to understand. This different perspective has often been characterized as the distinction between the exterior and the interior perspective [59]. The natural sciences look at an object from the outside. Their aim is to observe and describe the nature and function of this object [60]. In order to understand the mechanisms which drive human and non-human life, they create empirical designs in order to prove the validity of their hypotheses. The humanities, by contrast, focus on an internal perspective [61]; they are interested in introspection, in matters of the human mind. This has often been described as a form of speculation, a concept which, in this context, should not be taken to dismiss the truth-seeking potential of the humanities. Rather, the humanities have been concerned with their own specific methodologies and their own way of description and interpretation of what constitutes knowledge itself. The concept of epistemology is key in this context: The natural sciences set out to understand and explain the world. By contrast, the humanities ask what it means to know in the first place. Epistemology is hence the study of knowledge itself [62,63]. Whereas the natural sciences were seen as the science of description and explanation, the humanities were characterized as the study of understanding and interpreting. Unlike the (external) objects with which the natural sciences were concerned, the objects that the humanities sought to understand were themselves products of the human mind. This difference in approach may well be one of the reasons why today, new methodologies such as Pharmaceutical Humanities are so fruitful: they enable us to understand the human condition—including challenges to human health posed by diseases, environmental pollution, or political unrest—both from the outside and the inside.

One of the central questions that we are faced with today is whether the distinction between the natural sciences and the humanities, which C.P. Snow had once called “two cultures” [41] and which may be mutually incompatible, has become obsolete. In this context, researchers have stressed the need to revisit the nature–culture divide [64]. As sciences are concerned with uncovering the truth about human behavior, natural sciences and the humanities have a common goal, which can be defined as the basis of science itself: they seek to provide solutions to a given problem. To use an example: Human behavior is rooted in biology. Yet, the specific manifestation that human behavior takes constitutes culture [65]. Biology and culture are hence inextricably intertwined.

At the beginning of a new millennium, the problems that sciences and the humanities are called upon to explain and solve have become more complex than ever. New methodologies and technologies have been developed, which enable us to change what it means to be human to an unprecedented extent [66]. Technologically assisted reproduction provides new ways of coming into the world [67]. The methodology of CRISPR/Cas9 has made it possible to edit the human genome [68]. As particularly the case of CRISPR/Cas9 may demonstrate, however, the availability of new technologies does not in itself determine whether or how they can be used. The editing of the human genome has thus given rise to a multitude of ethical debates leading to suspending the clinical use of CRISPR/Cas9 [69,70,71]. As this example shows, the clear-cut distinction between the natural sciences and the humanities may lead us to an impasse here. The natural sciences seek to describe and explain human and non-human life; the humanities are interested in experience and introspection. In bioethical debates such as the one which surrounds the implementation of CRISPR/Cas9 in clinical practice, we need both perspectives. Here, the humanities can provide a form of orientational knowledge, which provides a compass or direction for future action [72]. New technologies may be made possible based on the knowledge developed by the natural sciences. How we should employ these technologies and what consequences they may have for human life, however, is a question that can be solved only through recourse to the humanities. The knowledge provided by the natural sciences and the humanities are mutually constitutive and need to be seen as complementary.

## 3. Pharmaceutical Humanities and the Notion of Paradigm Shifts

In light of these developments and the divergent historical trajectories which sciences and humanities have taken, we may want to consider the nature of scientific progress itself. When do new fields or methodologies emerge? What are the factors which lead to the development of new approaches? In order to gauge whether the current moment is ripe for the emergence and implementation of new fields such as Pharmaceutical Humanities, we must revisit earlier developments and the conditions under which, historically, innovation occurred. Two aspects are especially important in this context. First, there can be no interdisciplinarity without disciplinarity [70]. Interdisciplinarity can only emerge from perspectives which are firmly rooted in specific disciplines [73,74]. Any discussion of the emergence of so-called paradigm changes must necessarily be concerned with Thomas Kuhn’s study *The Structure of Scientific Revolutions* [75]. In his book, Kuhn addresses the question of innovation as such. He distinguishes between the constant strife for knowledge and innovation, which is inherent in any scientific and scholarly discipline, on the one hand, and, on the other hand, a form of innovation that is so fundamental that it challenges the discipline itself. For Kuhn, a paradigm is a form of knowledge that is shared by the practitioners of a given discipline; the very existence of paradigms, according to Kuhn, is constitutive to the formation of disciplines, as opposed to pre-disciplinary knowledge. Kuhn distinguishes two phases or currents within the history of science and in the course of scientific development. On the one hand, there is the *Normalwissenschaft* (normal science or standard science), a period in which knowledge in a specific discipline or a given field is consolidated and advanced within the parameters of this discipline. In Kuhn’s theory, these periods of *Normalwissenschaft* are disrupted through revolutionary phases in which paradigm changes occur [76]. The notion of the paradigm is central to distinguishing between these two forms of scientific development. In the former phase of the *Normalwissenschaft*, problems are solved according to the logic and the rules and assumptions inherent in a specific paradigm [77]. This has been compared to a game of chess: since the rules are given and are being shared by all players, innovation will occur only within this framework. Kuhn stresses the fact that innovation also occurs within this framework of *Normalwissenschaft*; yet, he goes on to specify that innovation will always remain within the structure determined by the paradigm in which problem solving takes place. According to Kuhn, revolutionary phases emerge under two conditions. First, a number of problems have occurred that cannot be solved from within the existing paradigm. Second, surprising discoveries have been made that serve to challenge this paradigm. At this point, it becomes necessary to question or challenge the rules of the game, to go back to the chess metaphor. The nature of the problems that need to be solved exceeds the possibilities that the rules of the game provide and hence calls for a change of rules, even of the game itself. This phase is conceived by Kuhn as a crisis situation [78,79]: all ground rules as well as the normal practice of scientific research in a given discipline have been unsettled. There is a need for a new paradigm to emerge, which will then once again be consolidated during a period of *Normalwissenschaft*. Unlike the phases of such standard or normal science, scientific progress in the time of paradigm changes is not cumulative: rather, innovation can be conceived more in terms of a push. In this as in other aspects, Kuhn’s theory is indebted to the work of Ludwig Fleck [80], who stressed that periods of scientific rupture are characterized by a change in scientific perception (*Wahrnehmung*) itself. One form of perception is hence superseded by another; the rules of the game have changed.

In this context, Kuhn’s theory of scientific revolutions differs significantly from the work of philosopher Karl Popper. Popper argues that a given scientific paradigm or approach will be abandoned only once it has been falsified [47,81]. Kuhn argues, on the other hand, that the abandonment of a disciplinary paradigm that has hitherto remained unquestioned does not occur solely on the basis of evidence.

Even as there are a number of aspects in Kuhn’s theory of scientific revolution that have subsequently been challenged, his approach nonetheless remains highly valuable, because it establishes a typology of scientific innovation. The key question that Kuhn addresses is the distinction between a change within a game and a change which is so fundamental that it challenges the ground rules of the game as such. A number of critics have taken issue with Kuhn’s theory. They have questioned, first and foremost, his use of the concept of the paradigm. Imre Lakatos, for instance, held that Kuhn’s idea of the paradigmatic is reductive since a paradigm could not be reduced to a single idea or conceptual definition [82]. It has also been critically discussed that Kuhn stressed the relevance of social factors for the emergence of new paradigms. Central to this and other critiques of Kuhn’s work has been the idea of constructivism. For Kuhn, scientific knowledge itself is part of cultural development. Human behavior may be the result of biological processes; yet, the specific manifestations of such biological processes constitute a cultural achievement [75]. This led physicist and Nobel laureate Steven Weinberg to the observation that for Kuhn science was akin to “democracy or baseball” [83]: Weinberg argued that in his “radical skepticism”, Kuhn reduced scientific progress merely to the fluctuations of social and cultural change. Similarly, Paul Feyerabend argued that Kuhn’s description reduced the sciences to a form of irrationality and unpredictable change [84].

These critiques notwithstanding, however, it could be argued that Kuhn’s theory of scientific progress remains central when it comes to addressing new knowledge formations, which exceed the boundaries of specific disciplines. At this juncture, it seems essential to recall the preconditions that, according to Kuhn, call for the emergence of new paradigms. Kuhn argues that new paradigms become necessary once a problem can no longer be solved from within one paradigm alone. The newly emerging field of the Pharmaceutical Humanities, if indeed it can be seen as a new field in its own right, must hence be able to address this very question: What problems would we be able to solve if we fuse the humanities and the natural sciences to create the Pharmaceutical Humanities? What questions would we be able to address, what blind spots would we be able to reveal, and what new solutions would we be able to formulate if a new, hybrid field were created?

In this context, we would like to return to a concept that loomed similarly large in Kuhn’s work on scientific innovation, namely the notion of incommensurability [85]. Kuhn suggests that a new paradigm can emerge when two previous paradigms, both of which have been consolidated through standard science in their given disciplines—a clash that is incommensurable. This would imply that two approaches exist that rival in being able to explain a specific problem or phenomenon. These two approaches, however, may be incommensurable because their ground rules, to return to the metaphor of the chess game, are fundamentally dissimilar. Because of this incommensurability, there can be no simple or smooth fusion between the two paradigms. Instead, a new paradigm must be created, which follows its own rules and is more than the mere sum of the previous paradigms. This notion of incommensurability is the element in Kuhn’s theory that has been the most controversially discussed [86]. Critics have argued that if these two prior paradigms were indeed incommensurable to the degree described by Kuhn, no dialogue between them would take place. With regard to the emergence of the potential new field of the Pharmaceutical Humanities, however, it could be argued that the notion of incommensurability can in fact be highly productive. So far, the burden of solving problems that are most pressing in the current moment—from the opioid crisis to the COVID-19 pandemic—has rested only on specific disciplines [87]. Problem solving has remained within the rules of a specific game, rather than asking whether the game is still sufficient to master this task. To return to Ludwig Fleck’s idea of the problem of perception (*Wahrnehmung*), each field will perceive the problem only according to its own logic. The logic of different fields may be incommensurable with that of the other. A crisis occurs, on the other hand, if the problem has become so complex that it can no longer be solved from within only one field. In order to perceive all facets of this problem, we must step outside the one field and view the same problem from the perspective of the other field.

Especially in the 21st century and with the increasing development of post-industrial societies, living conditions have changed considerably due to political, socio-economic, ecological, and cultural influences. In roughly the same period, the possibilities of medicine and pharmacy have shifted ever further in the direction of the influence of biotechnology and gene technology on health and disease as well as on human life processes (from reproduction and prenatal diagnostics to dying). There are new, spectacular options in dealing with the human genome (e.g., through genetic interventions into the human germline [88]), which can confront each and every one of us with unexpected choices, even in our everyday lives.

Such biomedical revolutions will inevitably be followed by profound changes at the societal level, which will alter our conception of the human being. This process can and should be accompanied by cultural and social sciences with different forms of life description (life writing). The convergence of these fields and their potential synthesis through complementary explanations of human life can become a crystallization point not only for the Medical Humanities but also for the Pharmaceutical Humanities, provided that these upheavals involve therapeutic and interventional measures on humans. In order to achieve this, common concepts must be established to bring the hitherto largely separate spheres of the cultural and natural sciences together more closely. This is essentially a matter of understanding the human body beyond its biological sense, since the body is always also a surface onto which cultural expectations, values, and societal demands will be projected.

As becomes apparent from these considerations, many of the emerging problems on our globe would be easier to solve in interdisciplinary working environments, rather than within individual scientific disciplines. As globalization increases, so do global problems. The more complex these threats become, the more urgent it becomes to think about cross- and interdisciplinary approaches to solving them. Here, the Pharmaceutical Humanities can provide a framework that facilitates the development of solutions that are otherwise difficult to achieve. This represents a new domain not only in the humanities but also in the basic biosciences and the pharmaceutical disciplines. These are some examples to illustrate threats that have become more pressing in recent times, and that might be addressed by Medical Humanities and Pharmaceutical Humanities:

- Warfare, nuclear threats, and genocides [89].
- Malnutrition and poverty due to overpopulation and overuse of agricultural or industrial resources of production [90,91]. They create a north–south divide on our planet that is already clearly visible, with waves of migration from the south to the northern hemisphere.
- Global warming and an imminent climate catastrophe [92].
- Environmental pollution control, development of sustainable production of goods, and the development of new high-tech recycling processes [93].
- Disease control and partnership-based access to standard medication for low- and middle-income countries [94,95].
- Research and development of new drugs for infectious tropical diseases (so-called neglected diseases) [96,97,98,99] microbial epidemics, and pandemics (i.e., malaria, tuberculosis, HIV, COVID-19, etc. [100,101,102]); development of rational alternative treatment strategies for primary health care in low- and middle-income countries [103].
- Fair access and benefit sharing for natural resources in developing countries. Implementation of the Nagoya Protocol and banning of biopiracy practices [103,104,105,106].

Are ways out of the crisis conceivable? In our opinion, the widely held political strategy of installing bans, laws, by-laws, etc., is suitable for achieving short-term and rather moderate success, but they are more like the proverbial drop in the ocean. In the long term, it will be essential to push ahead with technological developments in order to effectively combat the problems of mankind. New technologies have always been the motor for progress in the history of mankind, even if not always with positive consequences. Therefore, technological development must always be accompanied by a consideration of technological impact and risk assessment [107]. Here again, there is an intersection between natural sciences and the humanities. New technologies developed in industrialized nations must then be made available to emerging and developing countries, too, so that these countries can participate in economic development and prosperity. At the same time, measures must be taken to effectively combat corruption, as in some countries, this threatens the prosperity of broad sections of the population. Therefore, broad social acceptance must be created all over the world. Moreover, in saturated western societies, there is sometimes a somewhat technophobic tendency toward biotechnology and genetic engineering processes because of presumed risks [108]. It is a central goal of the Pharmaceutical Humanities to carry out necessary educational work in such contexts. At the same time, warnings must be issued about actual risks, and social discussion must be sought in order to make the right political decisions. An illustrative example of this is the so-called “gain-of-function” research, which is linked to risk-laden consequences that cannot be overlooked (with the risks even including biological warfare) [109,110]. All these aspects show the enormous potential for innovation through cross- and interdisciplinary research. The Pharmaceutical Humanities can become a hub of innovative research in the coming years and thus make a significant contribution to solving the most pressing problems facing mankind.

Such innovation through radical interdisciplinarity has already occurred in the newly established framework of the Medical Humanities. The problem addressed by the Medical Humanities, in its complexity, exceeded the perspectives and approaches that either medicine nor the humanities had to offer. In the course of an ever-increasing specialization of medicine, and with the ascent of biomedical technologies, some practitioners of medicine argued that the patient perspective seemed to have disappeared from view. They claimed that a countermovement was necessary, in order to return the practice of medicine to its original, humanistic goal [111]: to care for the individual in all their personal, social, biological, and cultural facets. However, it was argued that this multi-faceted approach to medical care at the beginning of the 21st century would be impossible to achieve from within medicine alone. Rather, there seemed to be a need to fuse medicine with the knowledge that could be provided by the humanities [6,112]. Central to this fusion was the patient perspective, as it had been expressed from times immemorial through literature and art. In the newly established fusion between medicine and the humanities, medicine and the humanities were no longer seen as incommensurable fields. Even as their disciplinary logic and their “game-specific rules” were vastly dissimilar, these two fields could nonetheless be combined. The notion of experience has loomed especially large in the Medical Humanities [113]. If medicine, in keeping with the logic of the natural sciences, sets out to describe illness and disease in objective terms by providing biomedical explanations for these phenomena, the humanities have been concerned with the experiences that these phenomena give rise to in the life worlds of human individuals [114]. In this context, it could be argued that each of these perspectives is incomplete without the other. As neuroscientist Wolf Singer has argued, knowledge formation, in the life sciences, has centered on a third-person perspective, the perspective of the impersonal and the objectifiable [115]. The perspective employed by the humanities, by contrast, is that of the first person; it is radically subjective and centers on the notion of individual experience. Each of these perspectives is incomplete without the other. For medical practice to exist, individual cases must be generalized into disease patterns; case narratives have to be generalized, and medical treatment has to be standardized. However, by stressing the role of the individual and the specificity of individual illness experience, the humanities—in the study of literature and art from patient perspectives—stress the role of the individual in the practice of medicine. This, as recent studies have shown, may be a key component in medical care and can serve to enhance doctor–patient communication [116].

## 4. The Emergence of the Pharmaceutical Humanities

### 4.1. Theoretical Foundations

The emergence of the Medical Humanities as a new field in-between the life sciences and the humanities can be located at a specific juncture in the development of both groups of disciplines. In the life sciences, the Medical Humanities can be said to arise from a “linguistic turn” in the life sciences, which has been highly influenced by Kuhn [117,118]. In the wake of this development, scientific knowledge production is no longer seen as being entirely separate from cultural and social developments, as has been outlined in Section 3. Rather, Kuhn’s constructivism argued that knowledge production in the sciences was itself a part of culture [75]. In the course of the linguistic turn in the study of knowledge production in the life sciences, studies emerged which stressed the role of language in conveying scientific discoveries. Lily Kay’s study of the rise of molecular biology as a lead discipline of the twentieth century may be indicative of this development [119]. Kay argues that this rise of a specific discipline to a power and a reach that it had not hitherto commanded was due to the metaphors that had been used in describing scientific discoveries in this field, especially with regard to DNA as the central element of life. Kay proposes that discoveries in this context were made during the Cold War; and the terminology in which these discoveries were described, and the metaphors that were being used, captured the mindset of Cold War societies. Crucially, Kay demonstrates in her study *Who Wrote the Book of Life?* that the advancements made in molecular biology at the time borrowed the vocabulary not of the natural sciences, but of information technology. Secondly, the description of DNA as the “book of life” invoked an imagination that was deeply rooted in Judeo-Christian ideas of the Bible being the book of life. This terminology, which was actually far removed from the realm of the natural sciences, served to enhance the power and radiant force that science communication in molecular biology was able to command both in their scientific communities and in the wider public [66].

The emergence of the Medical Humanities can thus be linked to the “linguistic turn” in the natural sciences. It is remarkable, however, that this linguistic turn in the life sciences was paralleled by a converging turn in the humanities. At the very time that the life sciences turned to language and narrative, the humanities took a material turn [120]. In the description of the emergence of new fields such as the Medical Humanities, this mutual convergence cannot be overestimated. This convergence centrally concerns the distinction between the natural sciences and the humanities as the so-called “hard sciences” and the “soft sciences”, respectively. The linguistic turn in the natural sciences, especially from those who have most adamantly criticized it, may have been seen as a “softening” of the natural sciences. Conversely, the allegedly ”soft sciences”, the humanities, were in search of the material reality that constructivist concepts seemed to have forgotten. This search for material reality which, regardless of the fact that this reality is accessible to us only through language, nonetheless exists outside language, also resulted in a renewed interest by the humanities in the logic of the natural sciences. Paul John Eakin, for instance, one of the leading scholars in the field of life writing research and autobiography studies, expressed his interest in the work of brain researcher Antonio Damasio [121]. Damasio, Eakin argued, might provide groundbreaking new insights into the way in which literary scholars trace the formation of memory in autobiographical and in literary texts [36]. In this context, one question seems to loom particularly large. Once a given cluster of studies has been identified as being part of a new research field or new paradigm, each paper published in this context may be seen in a different light. In other words, a new field or new paradigm may already have come into existence, before it is retrospectively identified as a new field. This question centrally concerns the idea of *Normalwissenschaft* or standard science formulated by Kuhn: When does a new field come into being, and when is it identified as such? And when does this new paradigm subsequently become standardized as *Normalwissenschaft*?

It is these considerations, which we will now translate into a discussion of the Pharmaceutical Humanities. It could be argued that the field of the Medical Humanities has been consolidated into an established new research field at the intersection of medicine and the humanities. However, as will be shown in the next section of this paper, a number of problems currently exist, which cannot be solved within the framework of the Medical Humanities. This particularly concerns the relationship between medicine and other disciplines within the life sciences. While within the life sciences, the role of medicine looms understandably large, there are other disciplines within the life sciences that may equally benefit from a dialogue with the humanities, and vice versa. To return to Kuhn’s foundational question, we may ask, what problems we may be able to solve by bringing together the knowledge production in pharmaceutical sciences (and pharmaceutical biology specifically) and in the humanities. It must be acknowledged here that the humanities, too, function as an umbrella term. The terminology of the humanities can be seen as paralleling the use of “life sciences” as an umbrella term for a very diverse group of disciplines, from internal medicine to molecular biology. However, it could also be argued that in order to grasp the logic of the formation of new scientific paradigms, it may be beneficial to explore the logic of specific groups of disciplines, as opposed to focusing specifically only on individual disciplines. As Wolfgang Frühwald, former president of the German Research Foundation (DFG), has argued, we might want to focus on what unites rather than what divides the humanities. The humanities, he suggests, are centrally concerned with all workings of culture. These workings of culture, Frühwald goes on to add, include the natural sciences. The humanities may hence be useful in that they explore, from the outside, the developments that take place in the development of the life sciences, even if they are not immersed in the specificity of their particular methodologies [122].

### 4.2. Results and Application: Examples of the Efficacy of the Pharmaceutical Humanities in Solving Key Problems of Our Time

As we have argued in Section 1 of this paper, new fields may come into being, if we are faced with problems so fundamental that they can no longer be solved from within one discipline alone, or even from within one group of disciplines alone. It is important to note that interdisciplinarity does not arise out of academic self-interest, but rather follows the requirements of problem solving. In the age of biomedicine and the life sciences, the problems that we are confronted with have become increasingly complex. Science and society must work hand in hand to find out not only what is scientifically possible, but also what is socially acceptable. While the life sciences have developed new technologies, the humanities and social sciences have focused on the implementation of these technologies in social practice [123]. In this section, we will map out a number of problems in which the interdisciplinary framework provided by the Pharmaceutical Humanities seems particularly promising: we select relevant topics from the literature providing ample evidence for the role of Pharmaceutical Humanities, and we select topics from our own research related to Pharmaceutical Humanities, as well as narrative pharmacy. As we will show, all these problems require problem-solving techniques from both the life sciences and the humanities.

#### 4.2.1. Selected Topics of Pharmaceutical Humanities from the Literature

##### Narrative Pharmacy

The requirement for narrative competence has been recognized in medicine, and narrative medicine is increasingly becoming an integral part of medical education. It is evident that narrative competence is also a key competence for the pharmacist, who is in constant contact with patients requiring medication [124,125]. Patients need to be advised on how to take their medication, they need to be informed about possible drug interactions upon comedication, etc. The compliance of patients to take their medication can be problematic and can considerably lower the treatment efficacy. The pharmacist has an important task to inform patients if they feel misunderstood, lost in the technocratic and sometimes anonymous medical system, or when they feel depressed about their disease. Patients seek help from unsound alternatives without letting their doctors know. Here the pharmacist may be helpful as a trustful person to talk to the patients. Although programs in narrative competence for pharmacists have been only scarcely established [126], the improvement of communication skills in pharmaceutical care effectively improves health care, and is, therefore, highly demanded on a routine basis in pharmaceutical curricula [127,128,129,130]. Narratives are not only helpful for patients but also for pharmacists to better understand the case history of patients and their individual disease experiences. The patients’ stories might also trigger an internal narrative in the pharmacist by reflecting and empathizing with the patient’s individual fate [131,132]. We investigated a case scenario demonstrating that patient narratives are not unilateral. Instead, they intertwine with the pharmacist’s personal thoughts and subjective impressions [133]. An overview of projects on the therapeutic role of narratives is shown in Table 1.

The role of narratives for therapeutic success can be illustrated from two different sides—from the view of the patient and the view of the healthcare professional. Although there is a large overlap of both aspects, we discuss published studies on narratives separately according to these two viewpoints.

A major issue in practical pharmacy is that many—if not all—elderly and comorbid patients take multiple drugs at the same time. It is by far not sufficiently investigated how diverse drugs interfere with each other. They may lead to non-intended synergistic interactions leading to increased drug activity. The contrary may also happen, and their activity might be diminished (antagonism), or if worse comes to worst, they may cause increased side effects and toxicities. The intake of multiple drugs is termed poly-pharmacology, and considerable concerns have been raised on this issue [134].

An observational study addressed the question on the value of narratives in this context [135,136]. The authors found that narratives of elderly, multi-morbid patients and caregivers can decrease the risk of inappropriate diagnostic investigations leading to subsequently reduced poly-pharmacological treatments. Hence, the risks of poly-pharmacology may be decreased by listening to the narratives of patients prior to drug treatment.

In addition to academic western medicine, various forms of complementary and alternative medicine raised attention, including traditional Chinese medicine (TCM). As previous studies found, narratives can improve the clinical efficacy of TCM [137]. Narratives improve diagnostics and therapy since patients’ attitudes and willingness can be helpful to modify and improve individual therapeutic interventions.

A number of studies have been published on cancer patients. Since cancer is potentially a life-threatening disease, the psychological problems of patients require special attention. Therefore, the role of narratives raised a lot of attention in clinical oncology. Illness narratives (e.g., written diaries) increased the empathy of healthcare professionals for patients [138]. Patients frequently suffer from sadness, fear, and loneliness due to pain, fatigue, side effects, and other cancer symptoms [139]. Narrating experiences or writing digital diaries during the time periods of chemotherapy improve not only the patients’ knowledge on their specific cancer type but also enhance their self-empowerment and communication abilities [140]. This result is important in light of the fact that still not all physicians discuss the imminent death of moribund cancer patients. From the experiences that have been published, the value of adequate conversations between the physician and patient is indispensable. The same is true for pharmacists. Either in public pharmacies or hospital pharmacies, the pharmacist can take over responsible tasks to talk to patients in progressed stages of their disease. An interesting project recently dealt with patient interviews after these patients’ reading two autobiographic books of other cancer patients [141]. These books served as a basis for intimate conversations between doctors and cancer patients. The unequivocal outcome of this project was that doctors should talk with patients about the nearly approaching death, and should listen to the patients’ emotions and prioritizations rather than to use medications that prolong the patients’ life span without noteworthy increase in life quality and in some cases even against the expressed will of the patient. Similar results have been reported in the palliative care of patients with end-of-life experiences [142]. In a cohort of 158 leukemia patients, written narratives supported the development of more sustainable healthcare services and therapeutic innovations to improve the quality of life and improve treatment compliance [143]. As a matter of course, the effectiveness of drug treatment depends not only on the efficacy and safety of the applied drugs but also on the compliance of the patients. Interestingly, a quantitative analysis of narratives dealt with the gender- and age-based drug compliance of cancer patients [144]. The evaluation of 257 patients revealed a better compliance of long-term-treated males than females and also a better compliance in elderly patients compared to younger ones.

The relevance of narrative also applies to other medical conditions. Prader–Willy syndrome and Ehlers/Danlos syndrome are hereditary diseases, where narratives can improve diagnostics and management [145,146]. Interviews and oral narratives improved clinical practice by supporting a better understanding of the daily life and social issues of patients.

The development of edema is a severe symptom appearing in diabetic macula. Oral narratives in the form of interviews with diabetic macula patients unraveled the difficulties to determine the optimal treatment and limited awareness [147]. Interviews may thus be a valuable tool for troubleshooting in order to improve healthcare of diabetic macula patients. Comparable results were reported for patients suffering from amylotrophic leteral sclerosis [148] and ventricular assist device patients [149].

Digital diaries have been studied for their suitability as illness narratives with therapeutic value. Epilepsy patients wrote digital diaries over a period of one year. Learning about patients’ experiences provided new information to the physicians, information that would otherwise have been lost. Diary writing should therefore be included in regular clinical practice, according to the authors of the study [150]. Migraine attacks can cause day loss experiences, as reflected by chaos narratives. A better awareness of these consequences of severe migraine should be implemented into clinical care [151].

In renal care patients, it has been observed that oral narratives not only improve the relationships between patients and caregivers but also disease management. The psychosocial experiences of patients represent an added value in nephrology [152].

**Table 1:** Effects of narrative medicine as non-interventional therapy on patients.

Topic	Hypothesis/Approach	Type of Study	Outcome	Ref.
Personalized treatment of elderly	Poly-pharmacology may be without efficacy	Observational study	Narratives of multi-morbid patients (over 75) and caregivers can decrease the risk of inappropriate diagnostic investigations and poly-pharmacological treatments (<10 drugs/day).	[135]
Poly-pharmacology	Narratives can help to reduce unnecessary drug intake	Randomized clinical trial	The evaluation of 604 patients from 55 primary care practices showed that improved doctor–patient dialogues reduce medication intake without affecting the health-related quality of life.	[136]
Traditional Chinese medicine	Narratives improve clinical efficacy	Comment	Narratives improve diagnostics and treatment because they highlight the patients’ emotions, willingness, and prioritization.	[137]
Oncology	Illness narratives increase empathy	Evaluation of interviews	Writing diaries creates empathy and greater intimacy. Professional skills in narrative medicine should be acquired by healthcare professionals and institutional organizations.	[138]
Oncology	Illness narratives increase empathy	Evaluation of written narratives	Patients suffer from sadness, fear, and loneliness due to pain, fatigue, side effects, and disease symptoms.	[139]
Oncology	Narratives improve patient’s awareness	Evaluation of written narratives	Writing digital diaries during chemotherapy improves knowledge on the disease, self-empowerment, and communication abilities of patients.	[140]
Oncology	Not all physicians discuss death with moribund patients	Evaluation of interviews	Interview with patients after reading two published autobiographical books of other cancer patients. Doctors should better discuss coming death than use medications that prolong life span but not quality of life.	[141]
Palliative care and end-of-life experience	Narratives improve quality of life	Literature review	Improvement of patients’ communication abilities and quality of life.	[142]
Oncology	Narratives improve healthcare and quality of life	Evaluation of written narratives	The evaluation of narrative diaries supported a better understanding of emotions experiences of 158 leukemia patients. They help to develop more sustainable healthcare services and therapeutic innovations to improve quality of life and improve treatment compliance.	[143]
Oncology	Gender- and age-based differences in drug compliance	Quantiative analysis of narratives	The evaluation of 257 patients showed better compliance of long-term treatment in males than in females and in elderly than in young patients.	[144]
Hereditary diseases	Narratives improve disease management	Evaluation of oral narratives	Patients with joint hypermobility syndrome/Ehlers-Danlos syndrome hypermobility type are frequently misbelieved and erroneously considered to suffer from psychatric or psychosomatic disorders. Narratives improve the patient-doctor dialogue, treatment and disease management	[145]
Hereditary diseases	Narratives improve diagnostics and management	Evaluations of interviews and oral narratives	Narratives help to understand the daily life and social issues of patients with Prader–Willi syndrome. Narratives help to improve clinical practice.	[146]
Amylotrophic lateral sclerosis	Narratives identify functional problems of patients	Evaluation of oral narratives and questionnaires	Narratives represent a main measure to personalize rehabilitation programs.	[147]
Diabetic macula edema	Troubleshooting to improve healthcare	Evaluation of interviews	Interviews of patients under intravitreal injection therapy and healthcare professionals unravel the difficulties for the best treatment mode because of complications and limited awareness.	[148]
Ventricular assist device patients	Narratives improve patients’ reflections	Evaluation of written narratives	Narratives act against loss of energy and improve patient’s autonomy. Narratives help to rediscover patient’s identity and improve resilience and quality of life.	[149]
Migraine	Illness narratives increase empathy	Evaluation of written narratives	Chaos narratives are linked to day loss experiences due to migraine. A better awareness of extreme experiences during migraine attacks should be implemented into clinical practice.	[151]
Nephrology	Added value of psychosocial experiences of patients	Evaluation of interviews	Oral narratives improve caregiver effectiveness and relationship between renal care patients and healthcare professionals.	[152]

Narratives can not only facilitate the psychologically fragile world of feelings of patients. They are a toolbox in the hands of therapists, who want to manage the fate of their patients in the best way. In this sense, narratives can be used as therapeutic measures. Therefore, we include here a chapter on the therapeutic viewpoint of healthcare professionals (Table 2) to complement the patient’s perspective outlined above.

This idea is not new: a review regarding the use of French post-war literature in narrative medicine concluded that narratives could pave new ways for healthcare professionals to think about the life of their patients [153]. An important goal of narrative medicine is to sensitize healthcare practitioners to the feelings and needs of patients. Hence, patient narratives cannot only be used to improve disease management for the sake of the patients but can begin one step earlier to better open the eyes of caregivers and to improve their empathy for the patients. As outlined in Table 2, a number of studies have been performed demonstrating that narratives as a non-interventional form of treatment can enhance the therapeutic competence. This has been shown in the education of students as well as professionals at all levels, and comparable results have also been reported from different countries (Italy, Korea, and other countries) [154,155,156,157,158,159,160,161]. Interestingly, training in narrative techniques did not only improve the competence of students in relation to the patients but also led to improvements in their academic achievements [154]. This may indicate that the training in narrative practice improved the motivation of students to learn more and to become better therapists [162].

Another comparative literature review focused on various non-interventional therapy techniques and discussed the value of narratives for treatment of somatic disease conditions. The authors concluded that the levels of evidence considerably vary among different methods of behavioral medicine and that understudied areas deserve to be investigated in more detail in the future [163].

**Table 2:** Generating narrative competence in healthcare professionals.

Topic	Hypothesis/Approach	Type of Study	Outcome	Ref.
French literature used in Narrative Medicine	Training of healthcare professionals	Literature review	French post-war literature opened new ways to think about life of patients.	[153]
Training of students	Illness narratives increase empathy	Clinical trial	Students of narrative medicine reached more empathic capabilities and reached better academic achievements.	[154]
Biopsychosocial connection	Harmonization of biopsychosocial consequences of biological diseases	Comment	Narratives influence physician–patient dynamics. Healthcare without empathy and compassion creates dissatisfaction among patients and healthcare professionals.	[155]
Training of health practitioners	Building multi-dimensional competence	Evaluation of interviews	Training improved awareness and medical service-based competence among healthcare professionals from Taiwan.	[156]
Narrative competence	Illness narratives increase empathy	Observational study	Medical students and doctors from Korea developed more empathy to patients by paying attention to patient narratives.	[157]
Narrative competence	Illness narratives increase empathy	Observational study	Patients, healthcare professionals, and family caregivers form an inclusive and empathic eco-system.	[158]
Writing digital diaries	Integration of narratives	Comment	The value of integrating digital diaries of cancer patients into clinical data files depends on the participation of the entire team of healthcare professionals.	[159]
Neuro-oncology	Illness narratives increase empathy	Evaluation of case-based histories	Narratives support the work of healthcare professionals by a better understanding of their needs and abilities.	[160]
Non-compliance to drug therapy	Compliance depends on doctor–patient relationship	Evaluation of written narratives	Clinicians realized that they should spend more time with patients to understand their motivations. This will improve drug adherence.	[162]
Behavioral medicine	Value of narratives for somatic conditions	Literature review	Comprehensive evidence exists for biofeedback, guided imaginary, and hypnosis techniques; less evidence exists for meditation techniques, disclosure therapies, and relaxation methods.	[163]

##### Pharmaceutical Education

Because the pharmaceutical curricula are filled with pharmacological and biomedical content, it is crucial to add topics from the humanities to strengthen the awareness of students and healthcare professionals for the special requirements of humanistic teaching content (Table 3).

A model curriculum has been developed in Japan. Experiments with this curriculum have been published [164]. The development of communication skills with patients was in the center of interest to increase the trust of patients to the pharmacist. Students were instructed to move their attention from pharmaceutical and medical-centered tasks to patient-oriented care by teaching them bioethical and medical-ethical topics as well as professional competencies in communication, therapy management, and interprofessional team care. Not only theoretical topics but also practical applications were trained. Despite broad agreement among the trainees about the usefulness of this training, there were also concerns that clinical and pharmaceutical practices could be still better interact with each other [165].

Another report deals with a seminar attempting to combine teacher- and learner-oriented approaches in the care of dementia patients. The outcome of the seminar and the students’ presentations with questionnaires highlighted the importance of empathy by caregivers for counseling and treating dementia patients [166].

The improvement is difficult to quantify and frequently a subjective judgement. Therefore, it is valuable that a recent study applied a psychometrical instrument, i.e., the Jefferson scale of empathy (JSE), to quantify the increase in empathy of pharmacists for their patients after participating in courses in narrative medicine [167]. In this study, 33 community and hospital pharmacists from Denmark participated in three courses. Patients filled out questionnaires on the pharmacists’ empathy before, during, and after the courses. The JSE scores were calculated for each participating pharmacist. The paired *t*-test was used and a statistically significant difference in the total JSE scores before and after the courses was calculated (*p* = 0.036).

##### Ethics in General and Practical Pharmacy

Ethics in general pharmacy and medical care are at the interface to governmental health policies. As such, several topics related to ethics and public health have been compiled in Table 4.

Ethical discussions may not only center around patients but may also consider issues related to the general public and the healthy population of a society. The production of medications and medicinal products cause carbon dioxide emissions as other production processes of any products also do. Pharmaceutical CO_2_ emissions represent a rather new and still neglected topic. In times of climate change, CO_2_ emissions are a tremendous problem in all fields of human life. Hence, ethics and other disciplines of the humanities shall also reflect on the detrimental effects of CO_2_ fingerprints in pharmacy [168]. CO_2_ emissions and environmental pollution occur not only during the production process itself but also during drug prescription and application (e.g., overprescription, development of antibiotic resistance, drug addiction, and lifestyle drug prescription). The final fate of drugs may also be critical for the global environment (e.g., trashing unused drugs by non-compliant patients, metabolization and excretion of drugs by patients, and waste of plastic syringes and other medicinal product waste harmful to the environment).

Another much-debated topic discussed in the public and politics of many industrialized countries is the use of cannabis for medical and recreational purposes. While some governments legalized its recreational use, others allowed even medical use only under clearly defined restrictions. From an ethical point of view, it is an obligation to develop competency for the medical cannabis use for both patients and healthcare professionals [169]. The need for recommendations for patients can be met by following other existing ethical principles of beneficence and non-maleficence and applying them to the use of cannabis. Guidelines for decision making by physicians and education of patients do exist.

Drugs that are not effective or are weakly effective for a fraction of patients but exert side effects are a long-recognized and critically discussed issue. During the past years, the concept of precision medicine emerged in an attempt to individualize drug treatment. The idea is to improve diagnostics and therapy in a way to be able to offer the right drug(s) to each individual patient. On the one hand, ethical discussions frequently appreciate that pharmacogenomic testing of patients may increase treatment efficacy and decrease side effects on an individual basis. On the other hand, genetic testing may identify people with inherited disease risks and predispositions [170]. As a consequence, this kind of information may not only be used for better treatment approaches may also include the risk of misuse (e.g., by the health insurance company or by employers) and may hence require careful risk–benefit estimations and legal regulations.

Drugs not used for disease treatment but for enhancement (e.g., doping in sports and lifestyle drugs) are ethically highly doubtful. The use of substances to improve body functions beyond normal levels has raised discussions among bioethicists and social scientists to better explore the “grey zones” facilitating the decision making and legalization by governments [171].

One of the best-known ethical issues is the conflict of interest. It is a problem if pharmacists and physicians interact inappropriately with the pharmaceutical industry. Conflicts of interest (i.e., monetary or non-monetary advances given to healthcare professionals by the pharmaceutical industry) may influence the outcome of the drug development process. Clinical phase I-III trials are required for the approval of drugs on the market, and the promotion of marketed drugs for routine application are processes that are sensitive to conflicts of interest [172]. The authors pointed out that the strong awareness of the conflict-of-interest problem in the medical and professional healthcare community may not always be beneficial but may to some extent also mask the view of other ethical issues.

##### Ethics in Clinical Trials and Clinical Pharmacy

The relationship between a doctor and a patient is characterized by trust. The doctor acts in the interest of the patient to cure them. Otherwise, medicine is not possible. If the doctor’s interests are prioritized over the ones of the patient, the patient’s interests are compromised. Therefore, it is an ethical duty to avoid conflicts of interest. Ethics has always played an indispensable role in medicine and pharmacy. There are a number of interesting studies highlighting that ethics is also important in the context of Pharmaceutical Humanities (Table 5).

An issue that is characteristic for many industry-driven drug development projects is the filing of patents for drugs. While the patenting practice was standard in the past century, manufacturers refused to patent their products before the 20th century. It was the American Medical Association dropping the prohibition of physicians holding patents leading to the situation we are facing nowadays. In a historical survey aiming to enhance the sensitivity of healthcare professionals, it was critically discussed whether pharmaceutical patents prioritize profits over advancements in medical care [173].

Another striking example is the opioid misuse scandal in the US. A recent public health analysis emphasized harm reduction as a main cause to facilitate the detrimental development of the opioid crisis. Harm reduction advanced many features that seem to be of advantage at first sight, e.g., promoted autonomy and well-being of patients. It promoted health equity, addressed racial disparities, and served vulnerable, disadvantages populations [174]. This striking contrast illustrates that a balance is needed.

The use of placebo treatments is a well-established state-of-the-art technique in high-quality clinical trials worldwide. However, there are also concerns on the use of placebos not from the perspective of drug approval by the pharmaceutical industry but from the viewpoint of the patient. In a recent review of studies, the authors argue that most arguments in favor of placebo use in clinical treatment trials could be refuted and are rather weak or even incorrect [175]. As pointed out in another biopsychosocial survey, placebo treatment may be in general morally acceptable but may involve deception and a violation of patients’ autonomy under certain circumstances [176].

The improvements advanced by the Pharmaceutical Humanities could be critically considered in a similar manner to other medical practices. Integrative approaches are needed where legal measures together with classical pharmaceutical and medical treatments are merged in a holistic manner for the patients’ sake.

##### The Relevance of Literature and Fine Arts for Pharmacy

It may be a bit unexpected at first sight how fine arts and literature can contribute to the treatment and cure of patients. However, there are a number of studies highlighting their value for the healing process (Table 6). This is not only true for patients but also for healthcare professionals who train themselves to apply specific practices to patients.

The implementation of content from the humanities into biomedical curricula was reported in a systematic literature review [2]. The curricular offerings comprised topics taken from creative writing, theater, music, and visual arts. Unfortunately, the authors did not come to the conclusion that curricular implementation of these contents made a clear impact. The small number of participants, short-term intervention, and over-reliance on subjective assessment have been discussed as possible reasons. Further investigations are warranted to clearly substantiate the benefit of including fine arts and literature into educational programs in healthcare, medicine, and pharmacy.

A recent narrative review gave an overview on non-drug-based approaches such as visual art instruction [3]. Training of healthcare professionals improved clinical observational skills, professional team formation, and communication abilities. Another important result was that visual art instruction improved the empathy of healthcare professionals for patients.

The experiences made with a practical course integrating drawing in healthcare curricula has been described. This arts-based course was initiated to enhance the professional skills of caregivers. The aim was to improve the reflection of the patient as a whole rather than seeing him/her as a biomedical problem. Drawings bear the potential to more easily obtain insight into the patient’s perspective and encourage creative and more holistic thinking regarding the nature of illness, the patient’s priorities, and the value of learning from the patient. The authors discussed possibilities to use drawings in an educational context [177].

Another interesting project dealt with the drawing of comics as a tool to cope with stress and burnout situations among students. An outcome of this extracurricular practical course was that comic drawing externalized stressful experiences and created a trustful atmosphere among the participating biomedical trainees [178].

Furthermore, after pharmacy students were introduced to methods from literature studies and cultural anthropology, they interviewed patients about their experiences with medication. A specific characteristic of this project was the application of a “close reading” approach. This is a text-immanent, hermeneutical mode of interpretation focusing especially on language style, comparisons, character constellation, and temporal structures as central features in narrative theory. This teaching project was accompanied by written reports, poster presentations, and two workshops to sensitize students to the patients’ experiences with medication, the central role of storytelling, and their professional role as pharmacists [179]. Interdisciplinary approaches including cultural anthropology and literature studies may hence supplement current pharmaceutical education to train communication skills in the practice of pharmacy.

#### 4.2.2. Selected Topics of Pharmaceutical Humanities from Our Own Research

##### Traditional Herbal Medicine

Indigenous knowledge of medicine has been gathered in all cultures and at all times worldwide. Mankind has taken advantage of the therapeutic power of medicinal plants since the very beginning of human evolution. During the past decades, fields such as pharmaceutical biology have been especially concerned with the role of traditional medicine as a means of improving primary health care in developing countries [180,181,182,183]. This development has led to a resurgence of interest in the medical knowledge held by indigenous groups around the globe.

What may be needed is thus an “[integrated] approach to health and medicine” in countries such as Africa [184]. For instance, in the treatment of HIV/Aids in countries such as Africa, a social approach to the health-seeking behavior of patients may be necessary. An approach may be needed to explore the social dimensions of patients’ lives. The exploration of the questions asked by the patients, such as “when, where and how to seek medical attention” [184] is not a trivial detail but may point to key aspects which need to be considered by both policymakers and healthcare providers. Patients’ narratives and their social circumstances are central when it comes to treating HIV/Aids in Africa, where there are limited resources and given the fact that a cure for the disease is still absent [184]. Patients may seek out traditional treatment options depending on their different backgrounds. For an optimal treatment to be possible, these attitudes and backgrounds need to be investigated. Where sociological studies have taken into account demographic factors and social structures, Pharmaceutical Humanities can provide an in-depth exploration of patient narratives and the experience they convey.

We started our investigations from the viewpoint of western medicine and expanded them to different kinds of “non-western” medicine. At this point, it is important for us to emphasize that we were not commercializing indigenous knowledge. Our aim is to assess diverse indigenous knowledge from traditional medicine with the same stringent scientific criteria as western medicine; not to demonstrate the superiority of western methods but rather, by contrast, to demonstrate the solid scientific and therapeutic basis of “non-western” approaches. The translation of indigenous knowledge into western scientific approaches and vice versa is convincing proof for us that century- or millennia-old forms of indigenous knowledge can indeed be recapitulated by western-style scientific methodologies [185]. Integrative medicine means that we should combine the best of “both worlds” for the sake of patients worldwide [186,187,188]. As the scientific discipline aiming to combine both approaches, ethnopharmacology integrates ethnobotanical knowledge with anthropological research [189,190,191]. During the past two decades, we performed more than 30 ethnopharmacological projects based on topics from Asia, Africa, Near East, and South America, e.g., [192].

Researchers have set out to determine with western laboratory methodologies the medical efficacy of substances used in traditional medicines—from indigenous medicine to traditional Chinese medicine (TCM). Studies have also investigated the potential side effects of alternative and traditional medicine [193,194,195,196,197]. While it would seem that such research is based only in the realm of life sciences, it also has profound social implications. To the extent that scientists were able to confirm the medical efficacy of remedies that have been described in indigenous medicine as well as TCM, sometimes even for thousands of years, this had profound implications for how we understand the knowledge held by indigenous communities. From a western perspective, such knowledge, until very recently, has sometimes been dismissed as mere folklore or superstition [198]. The fact that these traditional remedies can now be scientifically proven in their effectiveness leads to a profound revaluation of indigenous wisdom as medical knowledge. Laboratory methods in the field of pharmaceutical biology have thus had far-reaching social consequences: in view of these laboratory results, the knowledge from traditional medicine now appears in a completely different light; far from being only a form of superstition, it is not only visible as solid (bio)medical knowledge but also subject to modern hi-tech developments [199,200,201,202,203]. Scientifically proven knowledge of medicinal plants reveals several important implications:

- Medicinal plants can be used in combination with standard medication to form an integrative medicine with the aim to decrease possible toxicities and side effects of western medicine and improve the quality of life of patients [204,205,206].
- Medicinal plants can be used for complex diseases that cannot be satisfactorily addressed by current medicine (e.g., cancer, mental disorders, neurodegenerative disorders, addiction, etc.) [207,208,209,210,211,212].
- Medicinal plants play an indispensable role in primary health care in low- and middle-income countries, where a majority of patients cannot afford the costs of western medicine. WHO has repeatedly reported that up to 80% of the population in developing countries rely on traditional medicine and has proposed a strategic plan for traditional medicine [103]. Obviously, medicinal plants have a lot to offer to western medicine. They are valuable components for a One World Medicine [213] to combine the best of western and non-western medicine for the sake of all human beings on this globe.

##### Fair Access and Benefit Sharing of Indigenous Knowledge on Medicinal Plants

This concept to use and integrate traditional medicine attracts special attention in light of the danger of unfair misuse of indigenous knowledge on medicinal plants. The idea of fair access and benefit sharing fell on fertile ground in the past years after decades of largely unprotected use and the commercialization of indigenous knowledge by pharmaceutical companies. The illegal and immoral misuse was termed biopiracy [214,215] in the 1990s by environmentalists and non-governmental organizations. Although the term has been criticized by scholars of jurisprudence [216], it is still used in public discussions to describe the patenting by pharmaceutical companies and commercial exploitation of indigenous knowledge without sharing the revenues with the tribes and communities concerned. The discussion about biopiracy is closely related to the question of ownership of knowledge [217]. It has been considered a continuation of colonialism and, as such, a manifestation of neo-colonialism [218]. It could be argued that the practice of biopiracy is in fact rooted in a disregard for indigenous knowledge as scientific knowledge. If the knowledge that has been held by indigenous groups for centuries is said to be mere folklore, then this provides a fertile ground for the appropriation of such knowledge by pharmaceutical corporations. In the field of traditional medicine, this has sometimes led to spectacular court cases. For instance, the pharmaceutical company Monsanto, now owned by Bayer, Germany, tried to take out a patent for the medical usage of turmeric [219]. Turmeric had been used in Ayurvedic medicine as an anti-inflammatory agent for thousands of years. Monsanto, however, set out to patent this knowledge by arguing that Indians had a cultural but by no means biomedical knowledge of turmeric. In a spectacular court case, however, the Indian government was ultimately able to prove the contrary: Hindu epics going back to ancient times could be taken as proof that this knowledge surrounding turmeric was not folkloristic, but biomedical in our contemporary usage of the term [220]. In this context, the full potential of the Pharmaceutical Humanities becomes apparent. In the pharmaceutical part of this interdisciplinary research, indigenous and traditional medicines are tested for their efficacy and their side effects. Even this scientific part of the research, however, requires researchers to immerse themselves in ancient texts: they must hence also immerse themselves into the cultural philosophy, which is inseparable from the medical knowledge contained in ancient scriptures such as the Bhagavad Gita. Moreover, the results of laboratory research can be translated into a new form of social acceptance of indigenous knowledge, which is shown to be biomedical knowledge. Finally, the Pharmaceutical Humanities are ideally suited to explore the difference between the economic gain of pharmaceutical companies, on the one hand, and the ethics of laboratory research, on the other hand.

Biopiracy does not only extend to the unauthorized use of indigenous or native knowledge of plants, but also encompasses the sampling of human tissue. Western biotechnological corporations have been eager to sample the tissue from populations such as the Tristan de Cunha Islanders, who have a significantly higher propensity for asthma. The aim of such tissue sampling is to study the genetic disposition of indigenous groups who are relatively small and homogenous. In keeping with the logic of biopiracy, such biotechnological practices have been termed “bleed and run” research—the western biotech company takes blood samples of native populations, who are not included in the benefits reaped through this research. Such tissue sampling can hence be seen as a commodification of human life [221]. In attempts to prevent such practices and to make them more equitable, companies can help these communities build new infrastructures that will benefit them in the future [222].

Pharmaceutical research can thus help to combat ongoing practices of biopiracy. This can be performed in a number of ways. First, research in pharmaceutical biology can prove that indigenous knowledge is biomedical knowledge, not mere superstition. Second, it can then go on to include indigenous researchers in the profit that can be made from such research. This can be accomplished, for instance, by including indigenous researchers as co-authors of scientific papers [222]. This shift is crucial: for far too long, the holders of medical knowledge in indigenous communities were only said to be native informers. Since indigenous knowledge was often dismissed as mere folklore, it was thought to be unnecessary to share potential profits made on the basis of such knowledge with the natives who had shared their information. Once indigenous knowledge is credited as knowledge in the biomedical sense of the term, however, this practice of native informants is revealed to be unethical. Here, the framework of the Pharmaceutical Humanities helps us to transform the role of the native informant into that of a fellow researcher [223], who must also be able to reap the benefits—economic or academic—of the research that is being conducted. Research thus conducted is a form of good practice both socially and scientifically: it is scientifically sound and socially equitable. Science and society are hence fundamentally connected in the search for new medical substances and can work in tandem to outlaw biopiracy. Efforts to counter biopiracy are also closely related to the protection of biodiversity [224].

Researchers need to reflect on the ethical implications of their research, taking into account different forms of social values. These values may differ for different agents (corporations, governments, and NGOs) and must be carefully weighed against each other [225]. Entrepreneurs may seek a herbal remedy for diseases such as HIV/Aids; NGOs, on the other hand, may be concerned with the preservation of indigenous knowledge. Researchers must hence be trained to be aware of the ethical implications of their studies, and the role of social value in these ethical considerations. Here, the Pharmaceutical Humanities can serve as a bridge which explores the narratives by these different stakeholders; it can contribute to the understanding of research ethics with regard to the study and use of traditional medicine.

As a research field which can effectively serve to resist practices of biopiracy, cultural biogeography can be of key significance in linking the “complex biogeographical and cultural history” of plants such as *Q. amara*, which is found in French Guiana [226].

It is particularly noteworthy here that the Medical Humanities have played a role in combatting biopiracy. In India, they have helped build the Indian Traditional Knowledge Digital Library (TKDL). The TKDL was built in 2001, after India had successfully fended off the patenting of turmeric, basmati, and neem by US-based biotech companies [227]. The aim of the digital library, akin to similar projects in Peru [168], was to prevent future cases of the patenting of traditional knowledge by western-based corporations. In contexts such as this one, the role of the Pharmaceutical Humanities could be the articulation of the complex interconnection between culture, knowledge, society, and pharmaceutical practice.

The World Health Organization (WHO) and the United Nations Educational, Scientific, and Cultural Organization (UNESCO) recognized the need to protect indigenous knowledge on the same terms as any other issue in intellectual property rights. As a result, the UN Declaration on the Rights of Indigenous People (UNDRIP) was launched by the UN General Assembly in 2007. Furthermore, the Nagoya Protocol on Access to Genetic Resources and the Fair and Equitable Sharing of Benefits Arising from Their Utilization to the Convention on Biological Diversity (CBD) defined protective rules for traditional medicinal knowledge and compensation in those cases where inappropriate patenting already happened in the past [104]. The European Parliament took up this protocol (EU, No. 511/2014) and passed it as a bill in 2014. Most but not all EU member states agreed to ratify the Nagoya protocol as law in their respective national legislations. As discussed at a conference on biopiracy in 2017 [228], current practices are still inclined to use indigenous knowledge and raw material to manufacture commercial products in industrialized countries without letting the low- and middle-income countries appropriately participate in areas where these resources come from [105,106].

##### Post-Traumatic Stress Syndrome

One of the central benefits of the Pharmaceutical Humanities also lies in working against the homogenization of the life sciences under the singular framework of the Medical Humanities. This can be illustrated in the treatment of post-traumatic stress disorder (PTSD). It can be argued that PTSD functions not only as a medical diagnosis but also as a social problem. In order to solve this problem on both these levels, there is a need for interdisciplinary research. PTSD can develop upon exposure to severe traumata for an extended period of time such as warfare experiences, natural disasters (e.g., earthquakes), or criminal violence (e.g., sexual abuse or kidnapping). This mental disorder is characterized by recurring flashbacks, hyper-arousal (e.g., anger or hypervigilance), nightmares, and avoidance or numbing of the traumatizing event. PTSD frequently impairs social life. First recognized among US-American Vietnam veterans, the American Psychiatric Association included PTSD in the *Diagnostic and Statistical Manual of Mental Disorders* in 1980 (*DSM-III*).

Brain areas responsible for emotional memories are especially affected, such as the prefrontal cortex, amygdala, and hippocampus [229]. Stress reactions by the hormonal hypothalamic-pituitary-adrenal (HPA) axis are dysregulated [230]. Single nucleotide polymorphisms (SNPs) in hormone-related genes in addition to epigenetic changes in the limbic-HPA (LHPA) axis have been associated with PTSD [231,232,233].

The current treatment strategies demonstrate only limited or moderate success and consist of psychotherapy (narrative exposure therapy, trauma-focused cognitive-behavior therapy, and eye movement desensitization and reprocessing) and pharmacotherapy (sertraline and paroxetine). Similarly, narrative exposure therapy (NET) can be applied [234], which can be connected to narrative medicine. In this context, the narrative reconstruction of PTSD is not limited to written memories in the form of diaries or autobiographies. It also comprises internet blogs, online videos, and even non-verbal forms of representation that mirror life-threatening situations. A good example is the US-American internet blog *PTSD and Me: True Stories from Military Veterans* [235]. In these cases, the humanities unravel existential border-line experiences documented as narratives through all these forms of life writing. Narrative can also serve as a counterbalance to objectifying approaches to PTSD. According to Benner et al., “framing PTSD as an objective disease state separates it from narrative historical details of the trauma”. In order to include the patient experience, narratives can be obtained through interviews with patients and caregivers, for instance [236]. In keeping with approaches from the Medical Humanities, art therapy has also been used for military service members affected by PTSD [237].

During the past decades, occurrences of PTSD have been on the rise. This has also been due to soldiers returning from war situations, such as the peace-keeping missions of the United Nations [238]. As military personnel return home from crisis situations, having experienced traumatic situations, which often involve the killing of civilians or the experience of being ambushed, individuals may have difficulties readjusting to civilian life. At the same time, both their relatives and society at large may have difficulties understanding the nature of PTSD as a disorder. At this juncture, the Pharmaceutical Humanities can provide solutions for living with PTSD on both a medical and a social level. Pharmaceutical research can develop substances for the treatment of PTSD; yet, they may be complemented by the humanities and social sciences, in order to understand how this medical treatment can be embedded in social practice. Here, narratives—in the form of patient accounts, testimonies, but also fictional texts—can be crucial, because they convey to the wider public what it means to suffer from PTSD. By being able to immerse themselves into a description of an individual diagnosed with PTSD, the public may come to be more sympathetic to such a person [239]. Their behavior, resulting from sudden flashbacks or anxiety attacks, may seem less frightening to their relatives or the social environment because its causes are being explained through narrative. As a result, social pressure on the individual suffering from PTSD may decrease; individuals may find more social acceptance of their condition. As a result, there may be a change in demand by patients in terms of treatment. Individuals may come to favor treatment options that involve long-term treatment, and they may be willing to consider alternative treatment options. Here, science and society work hand in hand: Social practice may have a direct impact on the treatment options that persons affected by PTSD consider for themselves. The potential of the Pharmaceutical Humanities hence lies in linking pharmaceutical research on remedies for PTSD to the social climate in which patients demand treatment methods. This demand may then also affect prescription policies and medical practice, even as it is in turn shaped by these policies and practices.

Mental disorders such as anxiety and depression are as old as mankind. Therefore, traditional medicine systems worldwide recommended herbal recipes that might be also valuable for PTSD treatment nowadays. In one of our projects, 19 plants from Chinese, Indian, and European traditional medicines were first identified on the basis of a literature review. Then, dichloromethane/methanol extracts of these plants were investigated for their antioxidant properties. Nine of the extracts with the highest antioxidant property were selected and tested on deficient *Caenorhabditis elegans* mutants for their stress-reducing properties. Subsequently, the chemical constituents of these extracts were identified by HPLC/MS. In particular, the substance ursolic acid was present in most of the extracts. In addition, withanolide A and withanone from *Withania somnifera* and ursolic acid showed antioxidant and stress-reducing effects in vitro and in vivo, possibly qualifying them as candidates for stress reduction [240,241,242,243,244,245].

Ancient TCM textbooks describe treatments for mood disorders such as *Xiao Yao San* (*Free and Easy Wanderer*) [246]. This herbal formula consists of Radix Bupleuri, Radix Angelicae Sinensis, white peony root, white Atractylodis, Poria, baked licorice root, fresh ginger rhizome, and mint herb. In the terms of TCM, this recipe relaxes the liver, nourishes blood deficiency, restores spleen weakness, and balances *qi*. It harmonizes the liver, spleen, and blood. Intriguingly, modern biomedical research confirmed the activity of such ancient recipes in preclinical network pharmacological investigations as well as randomized clinical trials. We reported that *Free and Easy Wanderer* significantly reduced oxidative stress through the KEAP1-NRF2/HO-1 signaling pathway and inhibition of NF-κB-mediated inflammatory responses [247,248]. In 2008, an earthquake with a magnitude of 8.0 on the Richter scale struck the Sichuan province in China. With an estimated 80,000 to 90,000 dead or missing and more than 370,000 people injured, this was a natural disaster of unprecedented dimension. We performed a systematic literature review and unraveled a high prevalence of PTSD [249]. A randomized, double-blind, placebo-controlled clinical trial on 245 survivors of this earthquake using *Free and Easy Wanderer* revealed significantly improved PTSD-related symptoms, i.e., improvement of somatization, obsessive-compulsive behavior, depression, anxiety, hostility, and sleep quality compared to placebo [250].

##### Opioid Crisis

One of the most recent problems in which the Pharmaceutical Humanities hold vast potential has been the so-called opioid crisis. In the US in particular, we have recently come to speak of an opioid epidemic, as more and more physicians prescribe opioid-based pain killers, and more and more patients ask for opioids in order to be able to function in the workplace [251,252]. It has been reported that in the year 2016 about 12 million Americans misused opioids and 64,000 died from overdoses [253]. This number is higher than that of the victims of the entire Vietnam war (1961–1975). Several factors account for this situation, e.g., questionable information on opioid safety, aggressive marketing strategies, the prescription practice of physicians, vulnerability of socially and economically disenfranchised groups, but also genetic predisposition. This clearly indicates that interdisciplinary approaches integrating life sciences and the humanities may be appropriate to tackle this problem. Through the framework of the Pharmaceutical Humanities, it can be shown that the opioid crisis is not only a medical but a social problem. What needs to be investigated here is not only the role of opioids in the treatment of chronic pain but also the prescription policy and patient demand [254]. Here, pharmaceutical biology can work as a mediator between medical practice on the one hand and the social understanding of chronic pain on the other hand. Moreover, Pharmaceutical Humanities can also intervene to investigate the role of pharmaceutical companies in this context.

Remarkably, the US-American Center for Disease Control (CDC) collected personal narratives about opioid addiction of patients or their relatives on its website [255]. Such personal narratives can help to understand the opioid epidemic and to make this problem and its risk factors known to a wider public. On the other hand, patient narratives may support the treatment of addiction [256]. Moreover, this example illustrates the essential role of health institutions like the CDC in fighting the opioid crisis.

As a number of studies have shown, doctors may feel pressured to prescribe opioid-based painkillers [257]. This may also lead to a failure to consider other forms of treatment for chronic pain, such as methods used in TCM such as acupuncture and herbal remedies [258,259]. At the same time, the interface between science and society is also important in the understanding of the opioid epidemic. As the pressure of the neoliberal workplace on the individual employee increases [260], patients suffering from chronic pain may ask their doctor for opioid-based painkillers, rather than asking their employer to reduce their working hours [261]. In this context, the Pharmaceutical Humanities would stress the fact that we need a social as well as a pharmaceutical remedy for treating chronic pain. The social remedy would inquire into the ever-increasing demands of the workplace in late capitalist industrialized societies [262]. Pharmaceutical research, on the other hand, would investigate both the role of economic interest in the widespread production and prescription of opioid-based painkillers and the potential of alternative and traditional medicine in treating chronic pain.

##### Takotsubo Stress Myocardiopathy (Broken Heart Syndrome)

Gender disparities have been described in many medical areas, including pathophysiology, disease progression, and treatment outcome. The underlying molecular mechanisms and social conditions in our communities are still incompletely understood but they are important to better devise strategies for the prevention, diagnosis, and treatment of diseases [263,264]. The example of the broken heart illustrates an astonishing co-construction of a narrative from literature and a clinical phenomenon. Intriguingly, this narrative becomes as important as clinical, genetic, and molecular data [265]. The potential of the Pharmaceutical Humanities also lies at the intersection between clinical practice, patient narrative, and pharmaceutical research. For a long time, heart attack among women was not recognized as a considerable problem of morbidity and mortality since heart attack is more common among men [266].

One example of the relevance of understanding the relationship between the social perception of a somatic condition, often conveyed through narratives such as patient accounts or literary narratives, can be found in the realm of cardiovascular disease. For decades, patients who had experienced the loss of a loved one had been describing the somatic effects of this loss. They had told their doctors that they think they might be dying of a ”broken heart”. This may sound like a romantic, sad love story. It is all the more surprising that there is actually a clinical phenomenon at the root of it. In cultural terms, the metaphor of the broken heart is one of the most common images to convey profound loss and emotional bereavement [267]. However, it is interesting to note that this widespread cultural usage was actually disadvantageous for medical practice. Until well into the 1980s, physicians underestimated the somatic nature of the disease: the metaphor of the broken heart actually proved an obstacle to the recognition and treatment of the broken heart as a disease. It was only in the 1980s that the so-called “broken-heart disease” was diagnosed as a somatic disorder. It could be shown that the experience of acute loss and bereavement can lead to a change in cardiac structure: a constriction that has serious consequences [268]. It was this change in the patient’s body that then gave rise to both the diagnosis and the official terminology for the disease: *takotsubo*, the medical term for broken heart syndrome, refers to a Japanese octopus trap. The shape of this trap—with a constricted entrance to a balloon-shaped container—refers to the change in the heart structure of patients suffering from takotsubo, or broken heart syndrome [269]. In this context, the role of the humanities is crucial: humanities research in the field of medicine and narrative can show that metaphors play a significant role in medical practice [120,270]. In this particular case of *takotsubo*, the use of a widespread metaphor (the proverbial “broken heart”) worked against the diagnosis of this syndrome as a biomedical condition.

Recent studies have found that the takotsubo syndrome can be triggered not only by sadness and grief, but also by happiness [271]. In both cases, the syndrome is inextricably connected with existential human experiences, which can also be expressed in narratives. Recent research has found that one of the earliest documentations of the takotsubo syndrome can be found in the New Testament, with regard to “the acute emotional stress associated with the deaths of Ananias and his wife Saphira” [272]. Researchers have also wondered whether Jesus died of a broken heart [273]. Questions such as this one indicate the immense interdisciplinary potential of bringing together life sciences and humanities: different narratives—from patient accounts to histories to the Bible—describe the experience of extreme emotions, which can then result in the takotsubo syndrome. Narratives, including popular culture, can also serve to disseminate knowledge about somatic disorders; popular figures such as the Grinch, who experiences his heart being “two sizes too small” [274] can thus be re-read through cardiological knowledge. Knowledge about how it feels to suffer from takotsubo syndrome can also be gained through patient narratives obtained in interview studies [274].

Clinically, stress cardiomyopathy is a transient left ventricular dysfunction that preferentially occurs in women (≈90%) after severe emotional stress such as the death of a child or husband, divorce, or other exceptional traumata as the causative trigger. Hyperstimulation of the heart is associated with markedly increased catecholamine levels and contractile responsiveness due to 2-adrenoreceptor and cAMP-mediated protein kinase A activation, which ultimately leads to myocyte injury and apoptosis. According to the American Heart Association, an estimated 1–3% of acute coronary cases are due to broken heart syndrome [268].

##### Global Environmental Pollution with Microplastics

Pharmaceutical biology and the humanities can also work hand in hand when it comes to combatting the effects of global environmental pollution. Marine plastic waste has formed several garbage patches in the Pacific and Atlantic oceans [275,276]. This is especially pronounced in recent research on the Great Pacific Ocean garbage patch. During the current COVID-19 pandemic, waste from face masks has exacerbated the “threat to the marine environment” [277]. At the core of the Pharmaceutical Humanities, there is the understanding that the life sciences and the humanities have their own “laboratories” for testing the effect that chemical substances have on the human body. The consequences of polluting our environment on a global scale have not been considered in the past and cannot be foreseen for the future. This is true not only for wildlife but also for human beings [278]. One aspect that has increasingly come into focus is that different chemical classes of plastic constituents exert estrogenic activity. This may have a considerable impact on human health as estrogen not only influences hormonal balance in healthy organisms but also contributes to carcinogenesis [279,280]. Breast cancer cells can be estrogen-dependent, i.e., they grow faster and in a more aggressive manner upon estrogen exposure. Plastic components that mimic estrogenic functions indeed foster the growth of breast cancer cells [281,282,283,284].

All organisms including humans have efficient detoxification mechanisms to deal with harmful xenobiotic compounds to maintain the integrity of their bodies. Among them are transport proteins in the outer cell membrane termed ATP-binding cassette (ABC) transporters that expel toxic exogenous compounds (e.g., taken up with food) from the cells. A major part of these xenobiotics (including microplastic compounds) is detoxified by these efflux pumps—a phenomenon termed “multi-xenobiotic resistance” [285]. Unfortunately, not all of these harmful compounds are extruded by ABC transporters, and some of these compounds even inhibit their efflux function leading to increased toxification rather than detoxification. The impact of these processes is not only evident for humans but for many other species as well, posing a huge problem for marine sea life [286].

However, such research may often fail to have a wider impact on the larger public in western societies. This is due to two reasons in particular. First, it has been the societies of the Global South, which have borne the most damage done by environmental pollution, particularly by the ocean garbage patch. Second, the question arises of how research in the life sciences can be translated to and circulated in the public sphere. In this context, forms of life writing—texts written by individuals, who have experienced the transformation of their own bodies due to plastic pollution—may be especially powerful. In other words, we may need concrete narratives of human experience to understand the danger inherent in plastic pollution. Seen from this perspective, these texts as reports of human experience can also be seen as a laboratory. Life writing texts—in the form of patient narratives, blogs, or autobiographies—also describe the effects of plastic substances on the human body, just as pharmaceutical biologists prove these effects in their own laboratories. In the framework of the Pharmaceutical Humanities, life sciences and humanities can thus work in tandem to investigate the detrimental effects of plastic waste on human health.

By taking into account individual and social consequences of environmental pollution, the Pharmaceutical Humanities can also show how environmental pollution and social injustice have been working in tandem [287]. Problems such as overpopulation, global warming, weather extremes, poverty, and chronic illnesses have affected some communities and countries more than others [288]. The concept of “environmental justice” is key in this context, since “environmental harm … disproportionately affects vulnerable demographic groups” [289]. The Pharmaceutical Humanities would hence link research in pharmaceutical biology about the negative consequence of plastic pollution on human health with research in the humanities about the social consequences of such pollution.

## 5. Conclusions and Perspectives

As we have shown in this paper, the Pharmaceutical Humanities, in tandem with the approach of narrative pharmacy, hold significant potential for addressing key problems which we are faced with at the beginning of a new millennium: from the Great Pacific Ocean garbage patch to combatting biopiracy to the current opioid epidemic. At a time when the life sciences are becoming more and more diverse, and the range of options provided by life sciences research increases, there is a need for diversifying interdisciplinary approaches.

This article has been concerned with the question of how new fields come into being. As we have stressed in this paper, new fields emerge, if problems have become so complex that they can no longer be solved by one discipline alone. Over the past decades, the Medical Humanities have emerged as one such new field at the intersection between science and society. However, we have argued here that the potential of interdisciplinary research can be increased if we take into account the disciplinary diversity within the life sciences. In this context, pharmaceutical biology seems particularly promising. Research in this field has mostly focused on translational research linking pharmacology and medical practice [290]. However, we suggest in this paper that translational research can also occur at the intersection between pharmaceutical research and social practice.

Human life constitutes innumerous aspects from different angles, as do diseases. The reductionistic perspective of diseases has undoubtedly led to the revolution of medical and pharmaceutical advancement mankind has witnessed during the past two centuries. Nevertheless, diseases still dogging human life and especially the complex multi-factorial diseases (cancer, neurodegenerative diseases, cardiovascular diseases, etc.) still cannot be satisfactorily cured today. This indicates that it may be advisable to broaden the scope of therapeutic possibilities from the classical reductionistic viewpoint to a more holistic one. As outlined in Table 7, the more holistic view of the humanities may be valuable to complement the reductionistic view in classical pharmacy and medicine to reach significant surplus in treatment successes.

As long as there are diseases that are incurable, an expansion of the current therapy concepts should at least be considered and discussed. Integrative medicine tries to combine the best therapy approaches from western and complementary medicine. A comparable approach might be conceivable for the Pharmaceutical Humanities insofar as this involves methodological approaches from the humanities. The goal is to explore and successfully apply the therapeutic potential of these humanities methods, especially for diseases that have not yet been satisfactorily cured.

Intriguingly, Pharmaceutical Humanities share similarities to medical systems that are different from the established western medicine system, e.g., complementary and alternative medicine (CAM). Here it can be helpful to compare the basic characteristics of western and complementary medicine with regard to their general approaches to diagnosing and treating diseases and to transfer them to pharmacy or Pharmaceutical Humanities. In this context, an important distinction is that academic western medicine follows the principle of reductionism, while eastern and complementary medicine systems view the patient holistically (Table 2).

Since the scientific methods of the humanities and social sciences are in a way better comparable to those of CAM than those of western medicine and pharmacy, it can be hypothesized that the supplementation of such approaches to established western medicine and pharmacy may improve the treatment outcomes of a number of so far incurable diseases. In our opinion this is a strong argument to establish a new discipline termed Pharmaceutical Humanities in addition to the already existing Medical Humanities.

For many years now, it has been recognized in academic western medicine that reductionist therapeutic approaches, despite their success, are obviously not sufficient to diagnose and treat some diseases with sufficient certainty. For this reason, new research concepts have been developed that not only consider a single disease-causing element in its smallest reductionist unit but try to grasp entire systems by combining many individual elements into a larger whole. This new discipline is called systems biology, and it recognizes that the human body is a complex, higher-level system to whose function many individual elements contribute. Systems biology is based on complicated biological measurement methods, the results of which have to be analyzed with the help of bioinformatics due to the sheer vast amount of data. In the field of pharmacology and pharmacy, network pharmacology has evolved from systems biology, which attempts to utilize systems biology findings from the comparison of healthy and diseased systems (cells, organs, etc.) to develop new therapeutic approaches.

In the same way that systems biology and network pharmacology are expanding the reductionist view of western medicine [312], it is time to develop the theoretical foundations of classical medicine and pharmacy and to supplement the classical teaching code with the new content of the Pharmaceutical Humanities. This view is based on the far-reaching value of the Pharmaceutical Humanities with its holistic view on medicine and pharmacy, while modern medicine and pharmacy nearly exclusively focus on a reductionistic approach. Both approaches have advantages and disadvantages. In an endeavor to integrate the best of both worlds (i.e., reductionistic and holistic approaches), the Pharmaceutical Humanities have the potential for the improvement of current treatment concepts in established medicine and pharmacy. It is precisely the dualism of different systems (i.e., reductionism vs. holism) that allows for a fruitful further development of medicine [313].

In the sense of comprehensive integrative medicine, the differences between reductionist western medicine and holistic eastern medicine have been transferred to other health care areas in the past [314]. This also opens up the opportunity to introduce other and novel integrative elements into medicine and pharmacy with the help of Pharmaceutical Humanities. Therapeutic improvements that can be developed with the help of new methodological approaches from the Pharmaceutical Humanities can offer valuable supplements to existing therapy guidelines and thus combine the best of both worlds.

A study in which a total of 203 accredited programs in Medical Humanities in the USA, Canada, and the UK were scrutinized shows that this concept is promising. The study showed that it was precisely the renowned universities with high-quality medical training that also had Medical Humanities in their study programs [4]. This means that the leading universities in medical education have recognized the value of Medical Humanities. Therefore, we believe that study programs in Pharmaceutical Humanities will similarly enrich pharmacy education.

In conclusion, we suggest that the Pharmaceutical Humanities constitute a fruitful terrain in which such translation between both worlds—the reductionistic, natural science-coined classical way of pharmaceutical sciences, and the holistic, non-drug-based humanistic approach—is possible. As the problems that we are faced with increase, so does the need for interdisciplinary solutions. It is to this need that the Pharmaceutical Humanities can respond by being attuned to the demands both of science and society.

## Figures and Tables

**Table 3:** Implementation of learning contents from the humanities into the pharmaceutical curriculum.

Topic	Hypothesis/Approach	Type of Study	Outcome	Ref.
Model core curriculum in Japan	Pharmacy students need to develop communication skills with patients to increase the trust of the patient to the pharmacist	Experience report	Outline strategies for students to learn bioethics and medical ethics and to develop communication skills.	[164]
Model core curriculum in Japan	From product-centered tasks to patient-oriented care	Experience report	Ten professional competencies (professionalism, patient-oriented attitude, communication skills, interprofessional team care, basic science, medication, therapy management, community health and medical care, research, life-long learning, education and training were investigated); however, there were concerns that clinical practice is not connected with knowledge of the humanities.	[165]
Dementia	Highlighting the patients’ and caregivers’ needs for empathy and counseling	Seminar	Combination of teacher- and learner-centered approaches using questionnaires and students’ presentations.	[166]
Narrative competence	Narratives improve empathy	Postgraduate course	Community and hospital pharmacists from Denmark. A statistically significantly higher level of empathy was observed after participating in three courses.	[167]

**Table 4:** Ethics in general and public pharmacy.

Topic	Hypothesis/ Approach	Type of Study	Outcome	Interdisciplinary Connectiveness	Ref.
CO_2_ emissions of healthcare industry	CO_2_ emissions of pharmaceuticals are understudied	Narrative review	CO_2_ emissions of pharmaceuticals are tremendous because of overprescription, antibiotic resistance, non-adherence, drug dependency lifestyle prescriptions, etc.	CO_2_ emissions are a common problem in life sciences and humanities	[168]
Medical cannabis	Ethical obligation to develop competency among patients	Comment	Developed recommendations are extensions of existing principles of beneficence and non-maleficence.	Development of guidelines for decision making and patient education	[169]
Precision medicine	Genetic testing for individualized drug treatment	Comment	Pharmacogenomic testing might increase the treatment efficacy and decrease side effects; identification of people with inherited disease risk.	Ethical guidelines are required for pharmacogenomic testing	[170]
Enhance-ment	Use of substances to improve body function beyond normal levels	Comment	Empirical engagement of resources for bioethicists to regulate new enhancements; outline of an empirical agenda for humanities and social sciences about enhancement.	Doping as extreme form of enhancement is illegal; “milder” forms are in a grey zone	[171]
Conflict of interest	Key issue in drug development and promotion	Comment	The discussion on conflicts of interest obscures the view to other ethical issues	It is an ethical issue if pharmacists and physicians interact with the pharmaceutical industry	[172]

**Table 5:** Ethics in clinical trials and clinical pharmacy.

Topic	Hypothesis/ Approach	Type of Study	Outcome	Interdisciplinary Connectiveness	Ref.
Patents	No prioritization of profit over medical advancement	Historical survey	Before 20th century: manufacturers refused to patent their products. During 20th century: American Medical Association dropped prohibition of physicians holding patents.	Ethical sensitivities of physicians	[173]
Opioid misuse	Harm reduction strategy	Public health analysis	Harm reduction promotes autonomy and well-being of patients. It advances health equity, addresses racial disparities, and serves vulnerable, disadvantaged populations.	Harm reduction to use drug injections	[174]
Placebo treatment in clinical trials	Placebos are well accepted among healthcare professionals	Review of survey studies	Most arguments in favor of placebo use can be refuted and are weak or incorrect.		[175]
Placebo treatment in clinical trials	Placebo may violate patient’s autonomy	Bio-psycho-social survey	The intentional use and exploitation of placebos and the placebo effect may be morally acceptable. However, under certain circumstances, there are concerns about the patient’s autonomy.	Ethical examination of clinical placebo use	[176]

**Table 6:** Relevance of fine arts and literature for pharmacy.

Topic	Hypothesis/ Approach	Type of Study	Outcome	Interdisciplinary Connectiveness	Ref.
Creative writing, theater, music, visual arts	Curricular implementation of arts and humanities	Systematic literature review	Unclear impact because of small sample size, short-term intervention, and over-reliance on subjective assessment.	Implementation of humanities into biomedical curricula	[2]
Visual art instructions	Training improves clinical skills and team formation	Narrative review	Improved development of clinical observational skills; promoted empathy, team building, and communication skills.	Training and education of biomedical students	[3]
Arts-based training for healthcare professionals	Integration of drawing in healthcare curricula	Practical course	Insights into patient’s perspectives: appreciation of (1) holistic impact of illness, (2) patient’s priorities, and (3) the value of learning from the patient. Reflection of personal holism rather than seeing the patient as biomedical problem.	Exploration of pathways to use drawings in an educational context	[177]
Stress and burnout among students	Comic drawing helps to express stressful situations	Practical course	Comic drawing externalized stressful experiences and created a trustful community among participants.	Extracurricular activities of biomedical trainees	[178]
Methods from cultural anthropology	“Close-reading” approach	Practical course	Students were sensitized to the patients’ experiences in their role as pharmacists.	Test-immanent, hermeneutic interpretation for pharmacists	[179]

**Table 7:** The reductionistic viewpoint in pharmaceutical sciences is complemented by the holistic approach in the humanities.

Reductionistic Approach	Holistic Approach
Dissects the system (body) in lower component levels [291]	Observes the body in its entirety [292]
Treats diseases at the lowest system level [293]	Treats diseases at the highest system level [294]
Explanation of diseases from inside to outside	Explanation of diseases from outside to inside [295]
Pathophysiological and molecular causes as treatment targets [296]	Restores internal (systemic) and external imbalances (lifestyle, environment) [297]
Role of treatment after onset of diseases by correcting disease causes	Role of prevention before onset of diseases by rebalancing internal and external factors of the human body
Evidence-based medicine [298]	Experience-based medicine [299,300,301]
Predominant method in western medicine (synthetic drugs, big pharma) [297]	Predominant method in eastern medicine (traditional Chinese medicine, Ayurveda), naturopathy, and western phytotherapy [302,303,304]
Patients’ thoughts and emotions are not essential for diagnosis and treatment [305]	Role of psychosomatic effects, role of patients’ mind and soul [306]
Role of the quality of a country’s health care system for the healing process [307]	Role of cultural background for healing process [308]
Reductionistic medicine may alienate patients [309,310]	Holistic medicine may foster patients’ understanding for their diseases [311]

## Data Availability

This study does not report original previously unpublished data.

## Abbreviations

ABC: ATP-binding cassette; cAMP, cyclic adenosine monophosphate; CBD, Convention on Biological Diversity; CDC, Center for Disease Control; COMT; catechol-O-methyltransferase; CRISPR/Cas9; clustered regularly interspaced short palindromic repeats/CRISPR-associated endonuclease 9; DSM, *Diagnostic and Statistical Manual of Mental Disorders*; EU, European Union; GABA, gamma-aminobutyric acid; HO-1, heme oxygenase 1; HPA, hypothalamic–pituitary–adrenal axis; JSE, Jefferson scale of empathy; KEAP1, kelch-like ECH associated protein 1; LHPA, limbic-HPA; NET, narrative exposure therapy; NF-κB, nuclear factor kappa B cells; NRF2, erythroid 2-like nuclear factor 2; PTSD, post-traumatic stress disorder; SNP, single nucleotide polymorphism; TCM, traditional Chinese medicine; UNDRIP, UN Declaration on the Rights of Indigenous People; UNESCO, United Nations Educational, Scientific, and Cultural Organization; WHO, World Health Organization.
